# EBDE clearing: A strategy for simultaneous 3D visualization and quantitative analysis of both renal arteries and glomeruli

**DOI:** 10.7150/thno.138951

**Published:** 2026-08-24

**Authors:** Yaping Wu, Qian Shen, Yan Liang, Lin Bai, Jinsai Wu, Qiuxiao Shi, Lei Wu, Menqi Wang, Yin Liu, Kui Huang

**Affiliations:** 1 Core Facilities of West China Hospital, Sichuan University, Chengdu 610041, Sichuan, China.; 2 Department of Radiology, The Affiliated Stomatological Hospital, Southwest Medical University, Luzhou 646002, China.; 3 Laboratory of Mitochondrial Metabolism and Perioperative Medicine, National-Local Joint Engineering Research Centre of Translational Medicine of Anesthesiology, West China Hospital of Sichuan University, Chengdu 610041, Sichuan, China.; 4 Department of Neurosurgery, West China Hospital, Sichuan University, Chengdu 610041, Sichuan Province, China.; 5 Department of Laboratory Medicine, West China Hospital, Sichuan University, Chengdu 610041, China.; 6 Department of Oral and Maxillofacial Surgery, The Affiliated Stomatological Hospital, Southwest Medical University, Luzhou 646002, China.; 7 State Key Laboratory of Oral Diseases National Clinical Research Center for Oral Diseases, West China Hospital of Stomatology, Sichuan University, Chengdu 610041, China.; 8 Luzhou Key Laboratory of Oral & Maxillofacial Reconstruction and Regeneration, Luzhou 646600, China.

**Keywords:** renal arteries, glomerular, 3D visualization, unilateral ureteral obstruction, diabetic nephropathy

## Abstract

**Background:**

Three-dimensional (3D) visualization of the renal vasculature plays a crucial role in the pathological assessment and mechanistic investigation of kidney diseases. However, existing 3D imaging approaches remain unable to rapidly and accurately achieve continuous visualization spanning from millimeter-scale arteries to micrometer-scale glomerular capillaries. Here, we developed an innovative strategy, termed EBDE clearing, which enables continuous 3D visualization from the renal artery to the glomerulus, systematically characterizes the spatiotemporal architecture of the entire renal arterial tree and glomeruli in unilateral ureteral obstruction (UUO) and diabetic nephropathy (DN) mouse models.

**Methods:**

We combined the vascular tracers of Evans blue and dextran with Ethanol-ECi tissue clearing (EBDE clearing) to achieve the improved labeling of all renal arteries and glomeruli. By further integrating light-sheet microscopy-based 3D imaging with reconstruction and segmentation, we performed high-resolution 3D visualization and quantitative analysis of both renal vascular and glomeruli in normal and diseased kidneys. Furthermore, we investigated, classified, and statistically analyzed the spatiotemporal architecture of the arteries and glomeruli in the kidneys of mice with UUO and DN.

**Results:**

Firstly, through quantitative analysis of renal artery diameter, vascular branching, and vascular segment straightness, we found that the arteries of both UUO and DN mice exhibited varying degrees of damage. The renal vascular damage in UUO mice was more severe, manifested by a significant reduction in vessel diameter and the number of vascular branches, as well as an increased in the segment straightness of some arteries; in contrast, the pathological changes in the renal arteries of DN mice were mainly concentrated in vessel diameter and the number of vascular branches. We further classified the glomeruli into three distinct types (M1, M2, and M3) based on their origins, and conducted a quantitative analysis of their 3D spatial distribution within the kidneys of UUO and DN mice, and discovered that in UUO mice, there was a severe reduction in the number and the average volume of the both total and the individual M1–M3 glomeruli. However, in the DN mice, apart from no changes in the total and M1 glomerular number, the other relevant pathological parameters of glomeruli also showed a decrease, although the extent of this decrease was much milder compared to that in the UUO mice.

**Conclusion:**

EBDE clearing enables continuous, high-resolution 3D visualization and quantitative analysis from the renal artery to the glomerulus in both normal and diseased intact kidneys. The study not only provides important insights into the renal pathophysiology of UUO and DN, but also establishes a rapid and comprehensive strategy for visualizing structural alterations during disease progression in the kidney and potentially other organs.

## Introduction

As the primary excretory organs of the body, the kidneys play a pivotal role in maintaining physiological homeostasis by regulating water, electrolyte, and mineral balance, while the renal vasculature is indispensable for these functions 1. The renal arterial tree exhibits a hierarchical branching architecture. Originating from the abdominal aorta and entering the kidney through the renal hilum, it sequentially branches into the main renal artery, segmental arteries, interlobar arteries, arcuate arteries, interlobular arteries, afferent arterioles, glomerular capillaries, and efferent arterioles, ultimately forming the post-glomerular capillary network, known as the vasa recta, thereby establishing an integrated intrarenal vascular system 2, 3. Vessel diameters range from approximately 300 μm in segmental arteries to only a few micrometers in capillaries 4, 5. Importantly, structural and functional abnormalities of the renal vasculature, including arteries, capillaries, and glomeruli, contribute to the pathogenesis of various kidney diseases, such as ureteral obstruction and diabetic nephropathy (DN). Glomerular injury may result in proteinuria, progressive renal dysfunction, and ultimately end-stage renal disease (ESRD) 6, 7. Similarly, ischemia and hypertension affecting the renal vasculature may contribute to renal dysfunction and chronic kidney disease 8, 9. Moreover, renal vascular rarefaction is closely associated with common cardiovascular and metabolic disorders, including diabetes, hypertension, and atherosclerosis 10. Therefore, elucidating the spatiotemporal organization, pathological alterations, and interrelationships between the renal vasculature and glomeruli is essential for improving disease diagnosis, therapeutic planning, and treatment response evaluation.

Over the past decades, ultrasound, magnetic resonance imaging (MRI), and computed tomography (CT) have substantially advanced the field of *in vivo* renal vascular imaging 11, 12. However, their application to high-resolution 3D imaging of the renal vasculature remains limited. For example, although photoacoustic imaging and super-resolution ultrasound imaging can visualize the renal vascular network, hemodynamics, and tissue oxygenation at a resolution of approximately 26 μm, these approaches remain incapable of providing detailed 3D structural information at the glomerular level 13, 14. Djonov and colleagues employed contrast-enhanced micro-computed tomography (micro-CT) to generate 3D images of the entire mouse kidney, spanning from the aorta to the glomeruli, with a local resolution of up to 2.2 μm. Nevertheless, this approach relies heavily on specialized micro-CT instrumentation, while passive perfusion-based imaging may fail to adequately visualize certain microvascular structures 15. Furthermore, functional MRI combined with specialized contrast agents has been widely applied to assess renal blood flow and renal artery stenosis. However, its spatial resolution remains insufficient for detailed visualization of glomerular capillaries 16-18.

Light-sheet fluorescence microscopy (LSFM) combined with optical clearing has increasingly been applied in kidney disease research, as it enables 3D visualization of tissue architecture at cellular resolution, thereby advancing our understanding of renal morphology and pathology 19-21. In recent years, numerous tissue-clearing methods have been developed to improve tissue transparency and imaging depth, thereby enabling comprehensive visualization of the renal vasculature. For example, Gunzer and colleagues employed anti-CD31 antibodies and lectin-based vascular labeling in combination with Ethanol-ECi tissue clearing to quantify glomerular number and size in whole kidneys 22, 23. However, due to insufficient labeling efficiency, large vessels—particularly arteries—were not adequately visualized. Dan Zhu et al. utilized a lipophilic dye (1,1’-dioctadecyl-3,3,3’,3’-tetramethy-lindocarbocyanine perchlorate, Dil) in combination with gelatin perfusion to improve vascular labeling integrity. Although this strategy enabled visualization of major renal vessels, glomerular structures were not adequately resolved 24. Another study combined dextran labeling with SHANEL clearing to achieve simultaneous 3D visualization of glomeruli and arterial vessels in human renal tissue 25. However, in our previous study, its performance in visualizing the overall renal vasculature in mouse kidneys remained suboptimal 26. In addition, transgenic mice combined with endothelial-specific vascular labeling and high-definition fluorescence micro-optical sectioning tomography (HD-fMOST) provide a promising approach for high-resolution visualization of the renal arterial network 5. Nevertheless, these approaches remain constrained by substantial limitations, including high cost, technical complexity, and prolonged experimental duration, limiting their broader applicability. Therefore, there remains an urgent need for a stable, efficient, and practical strategy capable of achieving high-resolution 3D visualization of the entire renal vasculature, encompassing both large arteries and microvascular structures, to facilitate investigations into vascular pathophysiological alterations in kidney diseases.

Building upon previous studies and extensive preliminary investigations, we found that combining Evans blue (EB) and dextran vascular tracers with Ethanol-ECi clearing—termed EBDE clearing—enabled comprehensive labeling of both renal arteries and glomeruli. By further integrating LSFM-based 3D imaging with image reconstruction and segmentation, we established a novel workflow for high-resolution 3D visualization and quantitative analysis of the renal vasculature and glomeruli in both healthy and diseased kidneys. Furthermore, we systematically investigated and quantitatively characterized the spatiotemporal organization of renal arteries and glomeruli in mouse models of UUO and DN. Through quantitative analyses of arterial diameter, vascular branching, and segment straightness, we observed varying degrees of vascular impairment in both UUO and DN mice. We further classified glomeruli into three subtypes (M1, M2, and M3) according to their arterial origins and quantitatively analyzed their three-dimensional spatial distribution in UUO and DN kidneys. In UUO mice, both the total number and average volume of glomeruli, including all M1–M3 subtypes, were markedly reduced. Collectively, our findings provide new insights into the remodeling of the renal arterial tree and glomeruli during the progression of urinary obstruction and diabetes, thereby improving our understanding of kidney disease pathology. Moreover, EBDE clearing provides an efficient and comprehensive strategy for 3D visualization and quantitative analysis of renal vasculature in the kidney and potentially other organs.

## Materials and Methods

### Animals and models

**Animals:** Eight-week-old (8 W) male C57BL/6J mice were purchased from Beijing Huafukang Bioscience Co and housed at the laboratory animal center of West China Hospital of Sichuan University. All animals were maintained in a specific pathogen-free environment with constant temperature and humidity (22 ± 2 °C with 55 ± 15% relative humidity; 12 h light-dark cycle). The mice were maintained in a specific pathogen-free mouse room under a 12 h light-dark cycle. All experiments and procedures in this study were approved by the West China Hospital of Sichuan University Medical Research Ethics Committee (Approval Number: 20250303057).

**Establishment of the UUO Mouse Model:** The UUO operation was performed as previously described 27. A small incision was made on the left flank of 8 W male C57 mice after anesthetization. The left ureter was ligated at two points using 4-0 silk, and the ureter was severed between the two ligations. Then, the muscles and skin were closed in turn. In the control group, ligation was not performed after the abdominal cavity incision. The kidney was collected 12 days after UUO surgery.

**Establishment of the DN Mouse Model:** Eight weeks old male C57BL/6 mice were fed high-fat diet (HFD) (Mediscience Ltd., Yangzhou, China), which contained 60% kcal fat. After 1 week of adjustable feeding and 4 weeks of HFD feeding, Diabetic mice were induced by a 5 days intraperitoneal injection of low-dose streptozotocin (STZ, S0130, Sigma) dissolved in sterile sodium citrate solution (pH 4.5). Blood samples were collected from the tail vein 5 days after STZ injection and determined using the glucometers (Sinocare, Shenzhen, China). Mice with random blood glucose values higher than 16.7 mmol/L were considered the successful establishment of the diabetic models. Diabetic mice continued to consume a high-fat diet for an additional 12 weeks after diabetes confirmation 28. Control mice were fed a standard diet and received equivalent volumes of 0.1 M citrate buffer. At the end of the study, the mice were euthanized under isoflurane anesthesia, and harvested the kidneys.

### Fluorescent dye perfusion for vascular labeling

To label the renal vasculature, glomeruli were labeled by medium-to-high molecular weight FITC-Dextran, and larger renal vessels were labeled with Evans Blue (EB). The working solutions were freshly prepared in saline at concentrations of 5 mg/mL for FITC-Dextran and 2 mg/mL for EB (Table S1).

The mouse was secured on a tail vein injection board. First, 50 µL of the FITC-Dextran solution was administered via intravenous injection, followed immediately by an injection of 50 µL of the EB solution. Successful EB injection was visually confirmed by the immediate appearance of a blue coloration in the mouse's paws and nose. After a 10 min circulation, the blood was removed from the mouse circulation via perfusion, and the kidneys were then harvested and fixed overnight at 4 °C in 4% paraformaldehyde (PFA).

We similarly labeled the renal blood vessels of mice with DyLight 488 Lycopersicon Esculentum (Tomato) Lectin (LEL-DyLight 488, DL-1174-1, Vector Laboratories) and Alexa Fluor 647 anti-mouse CD31 antibody (102416; BioLegend, Table S1) as previously described 24. A total of 100 μL of LEL-DyLight 488 (at a concentration of 0.5 mg/mL, Table S1) and 100 μL of Alexa Fluor 647 anti-mouse CD31 antibody (at a concentration of 0.2 mg/mL) were administered via tail vein injection. After 30 minutes of circulation, renal tissues were collected and fixed in 4% PFA at 4 °C.

After all tissues were fixed for 24 h, they were washed three times with PBS, with each wash lasting at least 1 h. The kidneys were embedded in 2% agarose and sectioned into 1mm thick slices using a specific mold (1 mm 40–75, RWD Life Science).

### Immunofluorescence and histopathological staining

After fixation, specimens underwent dehydration and paraffin embedding, followed by sectioning into 5μm-thick slices for immunofluorescence and histopathological staining. Sections underwent microwave antigen retrieval (pH = 8.0, high power for two cycles, 8 min each), and then were blocked in 5% goat serum for 30 min. Primary antibodies anti-CD31 (1:200, ab182981, Abcam) or Anti-alpha smooth muscle actin (anti-α-SMA, 1:300, ab5694, Abcam) were incubated overnight at 4 °C. Corresponding fluorescent secondary antibodies were incubated at 37 °C for 1 h. Nuclei were stained with DAPI (1:1000, D3571, TermoFisher) for 5 minutes. Sections were imaged using confocal microscopy. Besides, some paraffin sections were dewaxed and rehydrated prior to elastic fiber E.V.G. staining (Elastic Fiber E.V.G. Stain, Bio-Rad BA4083A) following kit protocols. HE, Masson (Masson trichrome staining kit, Sorlabio-G1340), and PAS (glycogen PAS staining kit, Sorlabio-G1280) staining were performed according to the kit protocols for each group.

### Tissue clearing procedure

For the CUBIC clearing method 29, we adjusted the experimental time and reagent exchange frequency based on previous research. The preparation method for the clearing reagent was completed according to existing formulas. The 1mm thick kidney slices were placed in 5 mL EP tubes, filled with CUBIC L reagent (Table S2), and incubated on a shaking table at 37 °C for 8 h, changing the reagent every 2 h. They were then washed three times in PBS, with one wash overnight, and finally immersed in CUBIC R reagent until the tissues became transparent (Figure S4).

For the FDISCO method (Figure S4), we followed the article published by Shan Zhu's team 30. The clearing process consists of two main steps: THF (Tetrahydrofuran) dehydration and DBE (Benzyl Ether) refractive index matching. We prepared solutions of 50%, 70%, 80%, and 100% THF in ddH_2_O and adjusted the pH of triethylamine to 9.0. Kidney slices were dehydrated in different concentrations of THF for 30 min, then in 100% THF twice until fully dehydrated; the dehydration process was carried out on a shaking table at 4 °C. Finally, they were placed in DBE solution until the tissues became transparent.

The method of Ethanol-ECi (Figure S4) 22, involved gradient ethanol dehydration (50%, 70%, 80%, 90%, 100%, 100% v/v), followed by refractive index matching in Eci (Ethyl cinnamate). The soaking time for each step was adjusted based on the size of the tissues: kidney slices were soaked for 30 min at each concentration, while whole kidneys were soaked for 2 h each time. Finally, in anhydrous ethanol, the tissues could be soaked multiple times until completely dehydrated. All steps can be performed at room temperature.

### Confocal imaging

Kidney tissue blocks and slices were directly placed on coverslips for confocal imaging (Nikon A1plus) using a Plan Apo VC 10×/0.45 DIC N1 objective. Z-stacks were obtained for the kidneys tissue blocks.

### Light sheet fluorescence microscopy-LSFM imaging

Whole kidneys were imaged using a light sheet fluorescence microscope. (Light sheet 7 Microscope, Zeiss, Germany) equipped with an EC Plan-Neofluar 5×/0.16 (Medum n = 1.529) objective, zoom 0.4. The acquisition mode settings were as follows: scan mode = frame, frame size = 1800 × 992 pixels, 2.29 × 2.29 × 6.04 μm per Pixel, bit depth = 16. z-Stack mode (define lower and upper limits) and tiling mode (2 × 6) were activated. Then, the light-sheet thickness was set to 12.08 μm, and dual illumination was used. After completing one round of image acquisition, the sample was rotated 180°, and imaging was repeated using the same protocol. For the glomerulus imaging channel, the excitation wavelength was 488 nm and the emission range was BP505–545 nm; for the renal artery imaging channel, the excitation wavelength was 640 nm and the emission range was LP660 nm. Raw data were obtained immediately after image acquisition, without spectral unmixing or crosstalk correction.

### Image processing

The raw files were imported into Zen blue software and stitched using the Stitching function. The z-stack images of both the anterior and posterior sides of the kidney were point-to-point aligned using the Fusion function in Arivis software. Finally, the images were exported as TIFF files and converted into IMS format for subsequent image processing.

### 3D visualization data processing and quantification

**Calculation of Fluorescence Signal-to-Background Ratio (SBR):** The calculation method referenced our previous article 26. Z-stack images at a depth of 200 μm from the surface were selected and processed into maximum intensity projections. Using the NIS-Elements BR software, fluorescence signals were subjected to threshold segmentation to obtain the average fluorescence intensity of the target signal. Regions of interest (ROIs) were drawn over 4-5 areas lacking the target signal to calculate the average background fluorescence intensity. The SBR was calculated by dividing the target signal intensity by the background intensity. The SBR was calculated separately for glomeruli and large blood vessels using the above method.

**Vessel Diameter**: Vessel diameter was measured via intensity profile analysis based on fluorescence signal. The vascular hierarchy (segmental, interlobar, arcuate, interlobular, and afferent arterioles) is classified primarily based on anatomical structure and vessel trajectory. We manually measured the diameter ranges of blood vessels at different grades. Vessels are categorized from the main trunk to the terminal branches, with vessel diameters decreasing progressively across categories. The diameter ranges for each level of the renal arterial tree differ between the normal and disease models. The specific vessel diameter ranges of three groups are listed below: 1. Control group. 1st: 224.2 ± 15.9 – 199.6 ± 8.5 μm; 2nd: 131.4 ± 16.9 – 99.6 ± 9.6 μm; 3rd: 87.4 ± 16.4 – 62.2 ± 11.4 μm; 4th: 44 ± 5.6 – 32.6 ± 5.0 μm; 5th: 31.6 ± 4.6 – 13 ± 2.9 μm. 2. DN group. 1st: 203.4 ± 9.9 – 177.8 ± 5.7 μm; 2nd: 108.8 ± 14.9 – 85.8 ± 7.8 μm; 3rd: 60 ± 4.8 – 48.2 ± 5.3 μm; 4th: 36 ± 3.9 – 29. 8± 1.8 μm; 5th: 22 ± 4.9 – 12.1 ± 1.8 μm. 3. UUO group. 1st: 158.8 ± 22.7 – 144.2 ± 23.3 μm; 2nd: 123.6 ± 19 – 109.4 ± 15.5 μm; 3rd: 52.6 ± 7.7 – 49.8 ± 10.3 μm; 4th: 28.4 ± 3.6 – 27.2 ± 3.9 μm; 5th: 23 ± 1.9 – 12 ± 2.1 μm.

**Vessel Branching and Straightness:** The vascular branches of interest were reconstructed using the "Surface" function (Table S3). The "Mask" function was then used to assign a value of 100 to signals inside the vessel and 0 to signals outside, creating a new vascular channel. The vascular structure was rendered using the "Filament" function (Table S4). In the analysis results, vessels were classified by diameter into main categories: interlobar arteries, arcuate arteries, interlobular arteries, and afferent arterioles. The number of branches for each category was counted. Each category was joined into a single segment, and the "Segment Straightness" parameter was used to obtain the straightness of the major vascular path. Following the classification of arterial vessels, five vascular paths were selected for each vessel grade (1st-5th) to calculate the average vascular straightness. The straightness for each grade was determined by the ratio of the straight-line distance between the path's start and end points to the actual distance along the path (Figure 2F). The specific calculation formula is as follows:



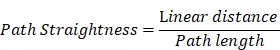



**Kidney Volume:** The total kidney volume was analyzed using Imaris 10.1 software. The outer contour of the kidney was manually traced using the "Contour" tool within the "Surface" function to create a rendered surface, from which the kidney volume was derived.

**Glomerular Counting:** The number and volume of glomeruli were analyzed using Imaris 10.1 software. The 3D structure of glomeruli was rendered using the "Surface" function. First, we utilized machine learning to segment glomeruli. Following 3D reconstruction, non-glomerular signals (primarily vascular structures in the medulla and kidney edges) were removed using a combination of surface area thresholding and manual verification (Figure S7). Based on a comparison of raw and reconstructed signals in Control and DN groups, signals with a surface area < 6000 μm^2^ were identified as non-specific and automatically excluded. Subsequently, we used manual screening to exclude a small number of non-glomerular signals. For the UUO group, to ensure the accurate retention of atrophic glomeruli and avoid size-based bias, all signals were verified via manual screening. The operator was blinded to the experimental groups during this process (Table S5). Finally, the number and volume data of the glomeruli were obtained. By selecting the "object-to-object" option between the glomerular and kidney surface objects, the quantitative distribution (count and volume) of glomeruli relative to the kidney surface distance could be obtained.

**Glomerular Length:** Glomerular length was directly measured from the glomerular basement membrane (GBM) boundary. For statistical analysis, 10 glomeruli in the renal cortex and juxtamedullary region were selected in every mouse, with a total of 3 mice included in the analysis.

**Classification and Statistical Analysis of Glomerular Origins:** Glomeruli were classified into three subtypes (M1–M3) based on vascular connectivity rather than spatial proximity, following the criteria described by the study 31. Specifically, we traced the vascular path of the afferent arterioles to categorize glomeruli into three groups based on the afferent arteriole generation sequence: Type 1 (2nd-3rd-5th), Type 2 (2nd-4th-5th generation), and Type 3 (2nd-3rd-4th-5th generation). Following this topological classification, we quantified the spatial distribution by calculating the number and volume of glomeruli located within a 200 μm radius of the identified afferent arterioles for each category (Figure S8).

### Statistical analysis

Statistical significance was assessed by independent-sample t test and one-way analysis of variance (ANOVA). All analyses were conducted with GraphPad Prism8. Quantified data are presented as mean ± SD.

## Results and Discussion

### The simultaneous visualization of renal arteries and glomeruli throughout the entire kidney

To establish a robust and reliable strategy for simultaneous tissue clearing and 3D imaging of renal arteries, we systematically evaluated two commonly used vascular tracers: anti-CD31 antibody and Evans blue (EB). Following tail vein injection and tissue clearing, their performance in whole-kidney vascular labeling and 3D imaging was comparatively assessed (Figure S1A). In anti-CD31-labeled kidneys, only glomeruli and a subset of renal arteries and afferent arterioles were detected, consistent with previous reports (Figure S1B) 22. This limitation may be attributed to the relatively short circulation time, which may have prevented sufficient antibody binding to endothelial antigens within the renal vasculature. Moreover, endothelial signals in large vessels were too weak to enable reliable visualization throughout the entire three-dimensional space. In contrast, EB enabled clear visualization of the entire renal arterial tree but failed to label glomeruli, capillary structures (Figure S1B), or veins (Figure S2), consistent with previous reports 20, 32. To investigate the specific targets of EB, immunofluorescence staining was performed on paraffin-embedded kidney sections. The results showed that EB fluorescence was predominantly localized to the arterial elastic fiber layer rather than vascular endothelial cells (anti-CD31) or smooth muscle cells (anti-α-SMA) (Figures S3A and S3B). Histological staining further confirmed that the distribution of EB fluorescence within renal arteries closely corresponded to elastic van Gieson (EVG) staining patterns (Figure S3C), which specifically highlight elastic fibers. These findings are consistent with previous reports demonstrating the high affinity of EB for elastic fibers and help explain why EB-labeled kidneys clearly revealed the renal arterial tree following tissue clearing 33-35.

Lectin and dextran are widely used fluorescent tracers for capillary labeling and have been extensively employed for visualizing glomerular capillaries in the kidney 23, 26. To identify the optimal tracer for glomerular labeling, the two aforementioned dyes were administered to normal mice, and their labeling efficacy for glomeruli was comparatively evaluated following Ethanol-ECi (ethyl-3-phenylprop-2-enoate (ethyl cinnamate)) clearing and 3D fluorescence imaging. Compared with lectin, dextran provided more comprehensive glomerular labeling together with a markedly improved signal-to-noise ratio (Figure S1B). Therefore, dextran was selected for subsequent experiments.

**Figure 1:**
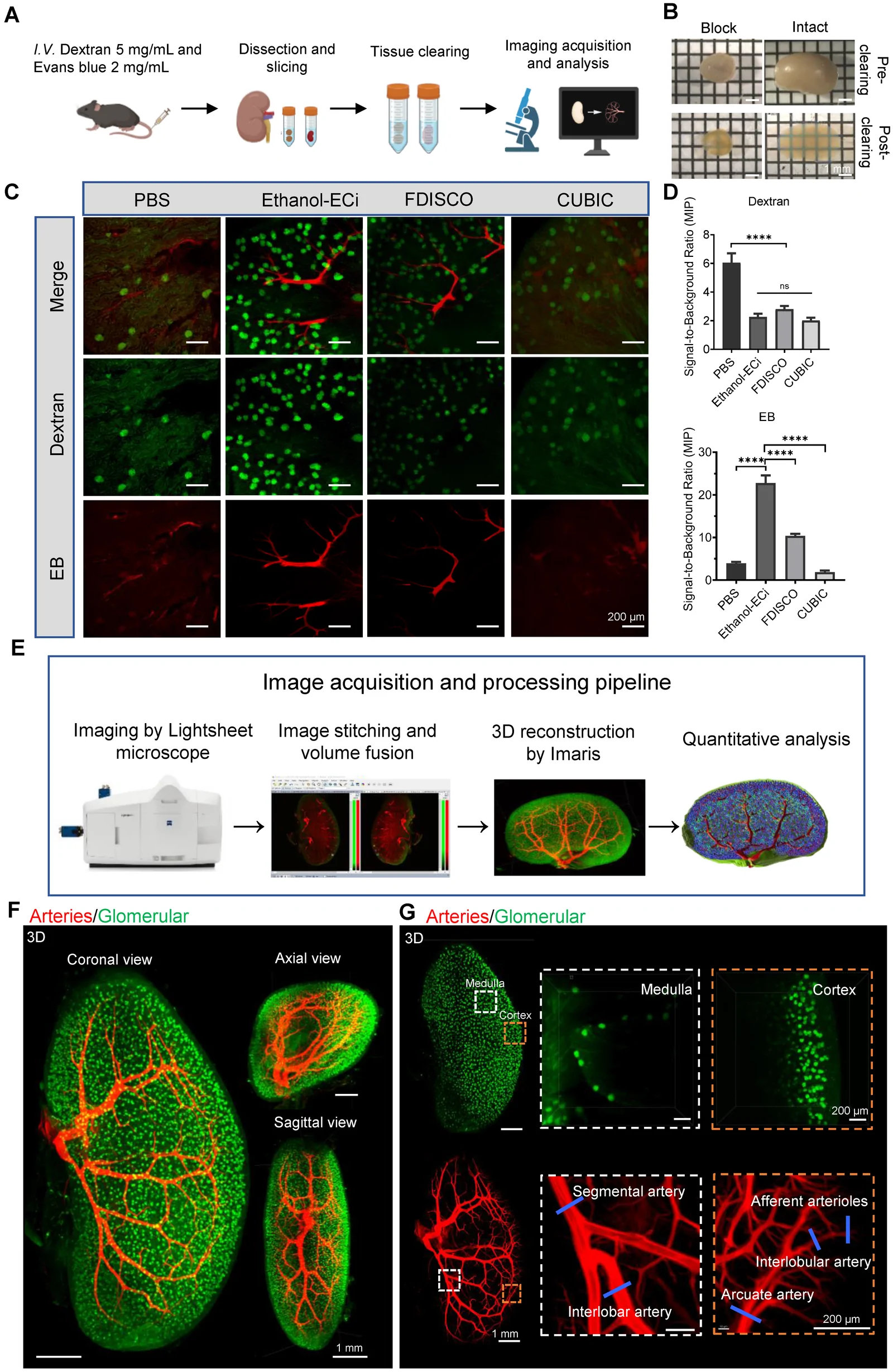
** EBDE clearing simultaneously enables a high-resolution 3D visualization of renal arteries and glomeruli in a mouse kidney. (A)** Experimental workflow for arterial labeling, imaging, and analysis. **(B)** The bright-field images of an intact kidney and a kidney block before or after tissue clearing using the EBDE clearing, scale bar: 1 mm. **(C)** The representative 3D fluorescence images of arteries in normal kidney tissue block labeled with EB and FITC-dextran after clearing with three different clearing methods (Ethanol-ECi, FDISCO, CUBIC). Green: glomeruli (FITC-dextran); Red: arteries (EB), scale bar: 200 μm, **(D)** Signal-to-noise ratios of labeled vasculatures using different methods, n = 6. **(E)** Image acquisition and processing pipeline for 3D vascular analysis of a normal intact kidney. **(F)** The 3D fluorescence images of overall arteries and glomeruli in an intact mouse kidney after tissue clearing by the way of EBDE clearing, glomeruli (green), arteries (red), scale bar: 1 mm. **(G)** The representative 3D reconstructed images for kidney glomeruli (green) and renal artery (red), and high magnification in different regions, scale bar: 1 mm and 200 μm. All values are presented as the mean ± SD. Statistical significance in **(D)** was assessed with one-way ANOVA, followed by the Bonferroni post hoc test. ****p < 0.0001, ns: no significance.

Based on these findings, we hypothesized that the combined use of EB and dextran, together with an optimized tissue-clearing protocol, could enable comprehensive three-dimensional (3D) imaging of the renal vascular network. To identify the most suitable clearing protocol, EB- and dextran-labeled kidney blocks were processed using several established tissue-clearing methods, including Ethanol-ECi, FDISCO, and CUBIC (Figure S4). The cleared samples were subsequently imaged using an inverted confocal fluorescence microscope, followed by quantitative analysis of vascular fluorescence intensity (Figure 1A and Figure S4). Among the evaluated tissue-clearing methods, Ethanol-ECi exhibited the highest signal-to-background ratio for both arterial vessels and glomeruli (Figure 1C, D). Therefore, EB and dextran were selected for labeling renal arteries and glomerular capillaries, respectively, in subsequent experiments. Normal kidneys were subsequently cleared using the Ethanol-ECi protocol (Figure 1B) and subjected to LSFM for 3D fluorescence imaging. The acquired images were subsequently stitched, reconstructed, and quantitatively analyzed using Imaris software (Figure 1E). Notably, the EBDE clearing strategy, which integrates EB- and dextran-based vascular labeling with Ethanol-ECi clearing, enabled simultaneous high-resolution 3D visualization of the renal arterial network across multiple hierarchical levels—including segmental, interlobar, arcuate, and interlobular arteries, as well as afferent arterioles—together with glomerular capillaries distributed throughout the renal cortex and medulla (Figure 1F-G, and Video S1).

### 3D visualization and recognition of renal arteries in an intact mouse kidney

**Figure 2:**
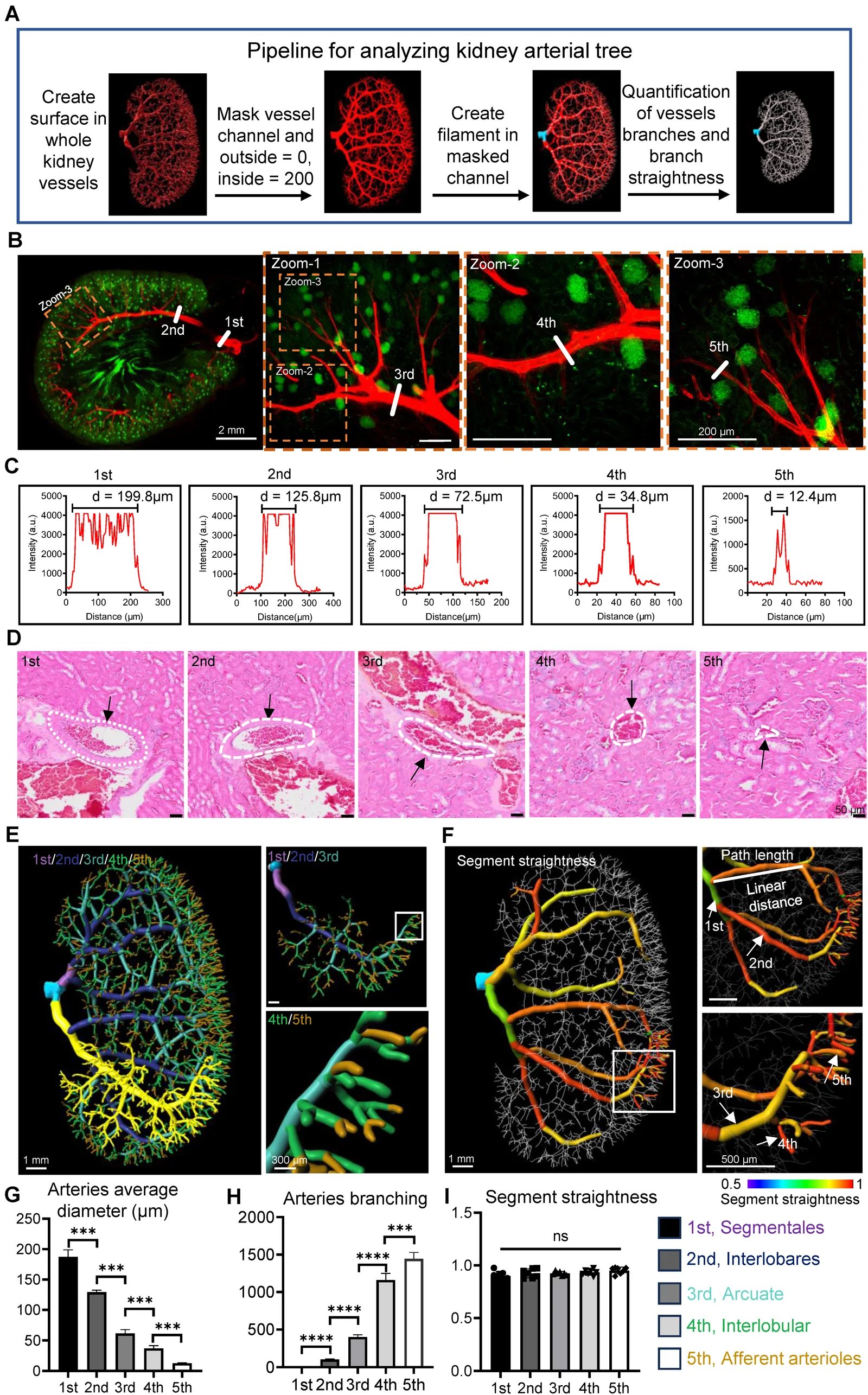
** The 3D visualization and recognition of renal arteries in an intact normal kidney. (A)** Schematic of the experimental workflow for analyzing kidney arterial tree using Imaris software. **(B)** Confocal images of kidney slice and its representative high magnification fluorescence images of kidney arterial vasculature at different branches. Green: glomeruli; Red: renal arteries, scale bar: 2 mm and 200 μm. **(C)** Intensity profile of arterial diameter at different branches of Figure 2B. **(D)** The representative H&E staining images of arteries of kidney at different branches. **(E)** The representative 3D rendering images of different vascular branches in a normal mouse kidney, scale bar: 500 μm. Purple: 1st, segmentales; Blue: 2nd, interlobares; Light blue: 3rd, arcuate; Green: 4th, interlobular; Yellow: 5th, afferent arterioles, scale bar: 1 mm and 300 μm. **(F)** Representative 3D reconstructed images for the vascular straightness from a normal, intact mouse kidney, scale bar: 1 mm and 500 μm. Quantitative analysis of the vascular diameter **(G)**, branches **(H)** and straightness **(I)** of an intact normal kidney, n = 5 (5 normal mice). All values are presented as the mean ± SD. Statistical significance in (C, D) was assessed with one-way ANOVA, followed by the Bonferroni post hoc test. ****p < 0.0001, ns: no significance.

As shown in Figure 2A, renal arteries were reconstructed and traced using the Filament Tool in Imaris software. Subsequently, we compared sensitivity of the automated and manual segmentation in reconstructing arteries and glomeruli from healthy and diseased kidneys using the surface tools in Imaris software, and found there was no significant difference between both (Figures S5 and S6). Therefore, in subsequent experiments, we chose the automated for arteries and glomeruli reconstruction and quantitative analysis. According to established anatomical criteria and vessel diameter, renal arteries were classified into five hierarchical categories 1, 3: Segmentales (1st), interlobares (2nd), arcuate arteries (3rd), interlobular arteries (4th), and afferent arterioles (5th) (Figure 2B). Compared with distorted arterial morphology observed in conventional histological sections, 3D fluorescence imaging enabled more accurate and objective visualization of the morphology and spatial distribution of renal arteries across all hierarchical levels, thereby facilitating precise quantitative analysis of arterial diameter, branching, and straightness (Figure 2B-D). Using this 3D vascular imaging strategy, the morphology and spatial distribution of renal arteries throughout the intact mouse kidney were clearly delineated across all hierarchical levels (Figure 2E, F, and Video S2). Quantitative analyses of arterial diameter, branching, and straightness across different vascular orders (1st–5th) revealed a progressive decrease in average arterial diameter with increasing vascular hierarchy (1st, 187.4 ± 10.17 μm; 2nd, 129.2 ± 2.99 μm; 3rd, 61.38 ± 5.53 μm; 4th, 37.26 ± 3.81 μm; and 5th, 12.76 ± 0.674 μm) (Figure 2G). Conversely, the number of arterial branches progressively increased with vascular hierarchy (1st, 6 ± 1.58; 2nd, 103.6 ± 7.99; 3rd, 401.4 ± 28.90; 4th, 1160.6 ± 88.05; and 5th, 1443.8 ± 86.14) (Figure 2H, J). However, there was no significant difference in the straightness of renal arteries across all levels in normal mouse kidneys (Figure 2I). Compared with the straightness variations of individual vascular segments, our statistical analysis of overall renal vascular straightness based on anatomical trajectories better reflects the global morphological changes of the kidney vasculature. It is worth noting, however, that this quantitative approach may overlook the localized changes within each individual segment. Furthermore, when compared with the 3D reconstruction results obtained via micro-CT 4, our labeling and imaging method achieves higher resolution in reconstructing the renal arterial tree (specifically for intact kidney datasets), along with shorter imaging times and higher success rates.

### 3D reconstruction and quantitative analysis of the glomeruli of kidney

**Figure 3:**
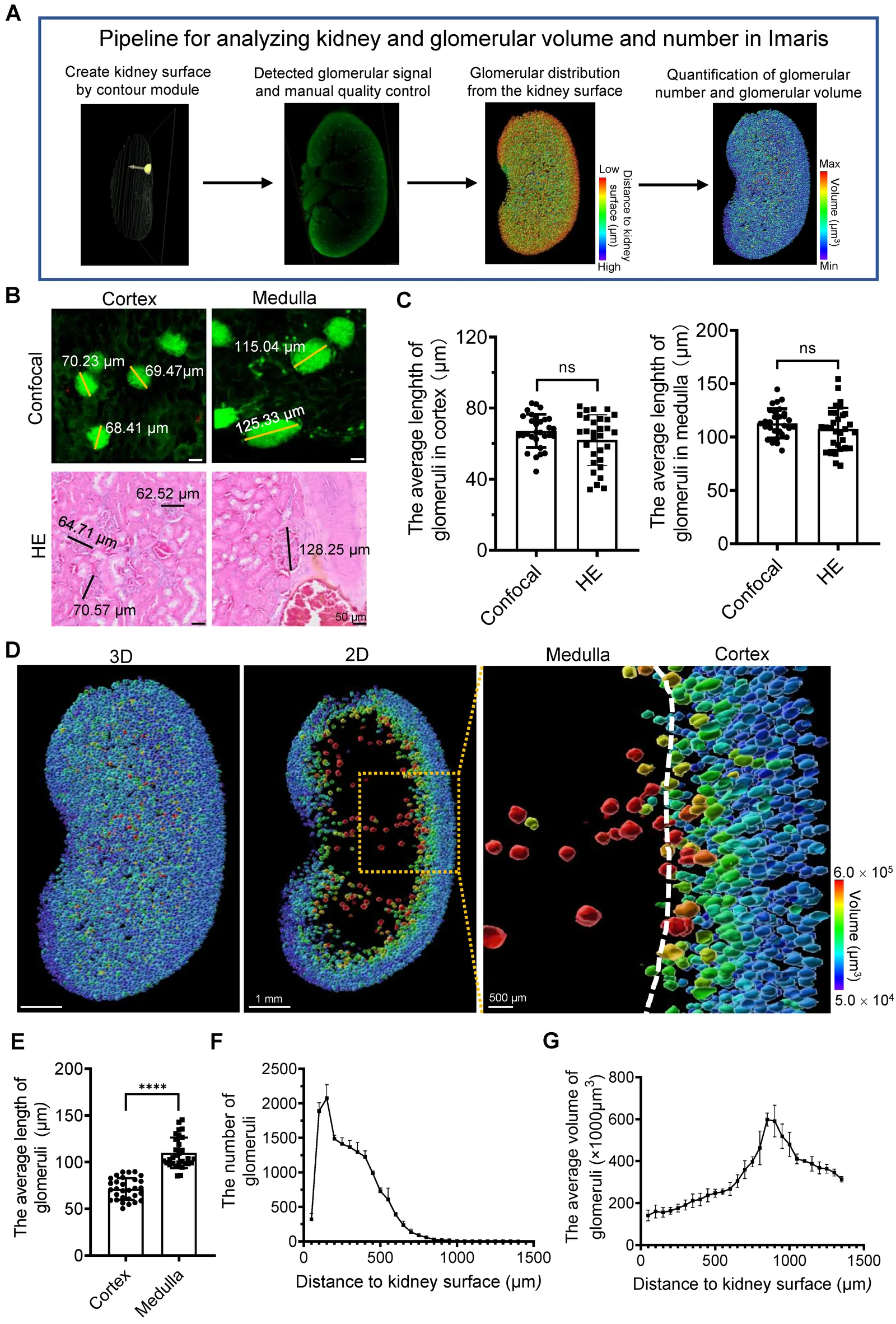
** The recognition of glomeruli of an intact normal mouse kidney. (A)** The workflow for analyzing kidney and glomerular volume and number using Imaris. **(B)** The representative fluorescence or light microscopic images of the renal medulla and cortical glomeruli of normal kidneys, following EBDE clearing or HE staining, respectively, scale bar: 50 μm. **(C)** Quantitative measurement and comparative analysis of glomerular lengths in the renal medulla and cortex of normal kidneys, following EBDE clearing + fluorescence microscopy imaging or H&E staining + light microscopic images, n = 30 (10 glomeruli per kidney, 3 normal mice). **(D)** 3D reconstructed images of glomeruli in the whole kidney or the largest kidney cross-section, scale bar: 1 mm and 500 μm. **(E)** The comparative analysis of glomerular diameters in the renal cortex and medulla of a normal kidney from Figure 3D, n = 30 (10 glomeruli per kidney, 3 normal mice). (F, G) Quantitative determination of the relationships between glomerular number, volume, and distance from the kidney surface, n = 3 (3 normal mice). All values are presented as the mean ± SD. Statistical significance in (C, D, E) was assessed with one-way ANOVA, followed by the Bonferroni post hoc test. ****p < 0.0001, ns: no significance.

The Surface Tool in Imaris software was used to identify, reconstruct, and segment 3D fluorescence imaging data of glomeruli (Figure 3A). Compared with conventional hematoxylin and eosin (H&E) staining, 3D imaging of normal kidneys following EBDE clearing demonstrated comparable accuracy in measuring glomerular length (Figure 3B, C). To further investigate the relationship between glomerular spatial localization and volume, all glomeruli within normal mouse kidneys were reconstructed and segmented, then visualized using a volume-based color scale. Within individual kidneys, glomeruli located in the superficial cortex (blue/green) exhibited smaller volume and diameter than those located near the corticomedullary junction (yellow/red) (Figure 3D, E, G, and Video S3). The precise spatial coordinates of each glomerulus enabled quantitative determination of the shortest distance between the glomerular center and the kidney surface (Figures 3D, F, G). Accordingly, detailed quantitative analyses of glomerular number and volume at different distances from the kidney surface were performed, revealing spatial distribution characteristics consistent with previous studies 23. The frequency distribution map revealed that most glomeruli were located within approximately 500 μm of the kidney surface, with peak abundance observed at a depth of approximately 250 μm, whereas only a limited number of glomeruli were detected beyond 1000 μm (Figure 3D, F). The average glomerular volume ranged from 100 to 600 × 1000 μm³, and initially increased with increasing distance from the kidney surface, reaching a peak at approximately 900 μm before gradually declining. Beyond 900 μm, the average glomerular volume decreased markedly with increasing depth, likely reflecting heterogeneous glomerular distribution within deeper renal regions. Glomeruli labeled with Dextran-FITC can achieve an imaging depth comparable to that of Lectin Dylight-647 23 and Alexa Fluor 647 anti-mouse CD31 antibody 22 using the Ethanol-ECi clearing, which offers significant advantages for multiplex tissue imaging. However, like other renal vascular markers, Dextran-FITC not only labels glomeruli but also other blood vessels, which may complicate the subsequent identification of glomerular signals. However, in this study, we effectively excluded non-target signals using manual segmentation based on their distinct morphologies and spatial locations.

### 3D visualization, classification, and quantitative analysis of glomeruli with distinct arterial origins

**Figure 4:**
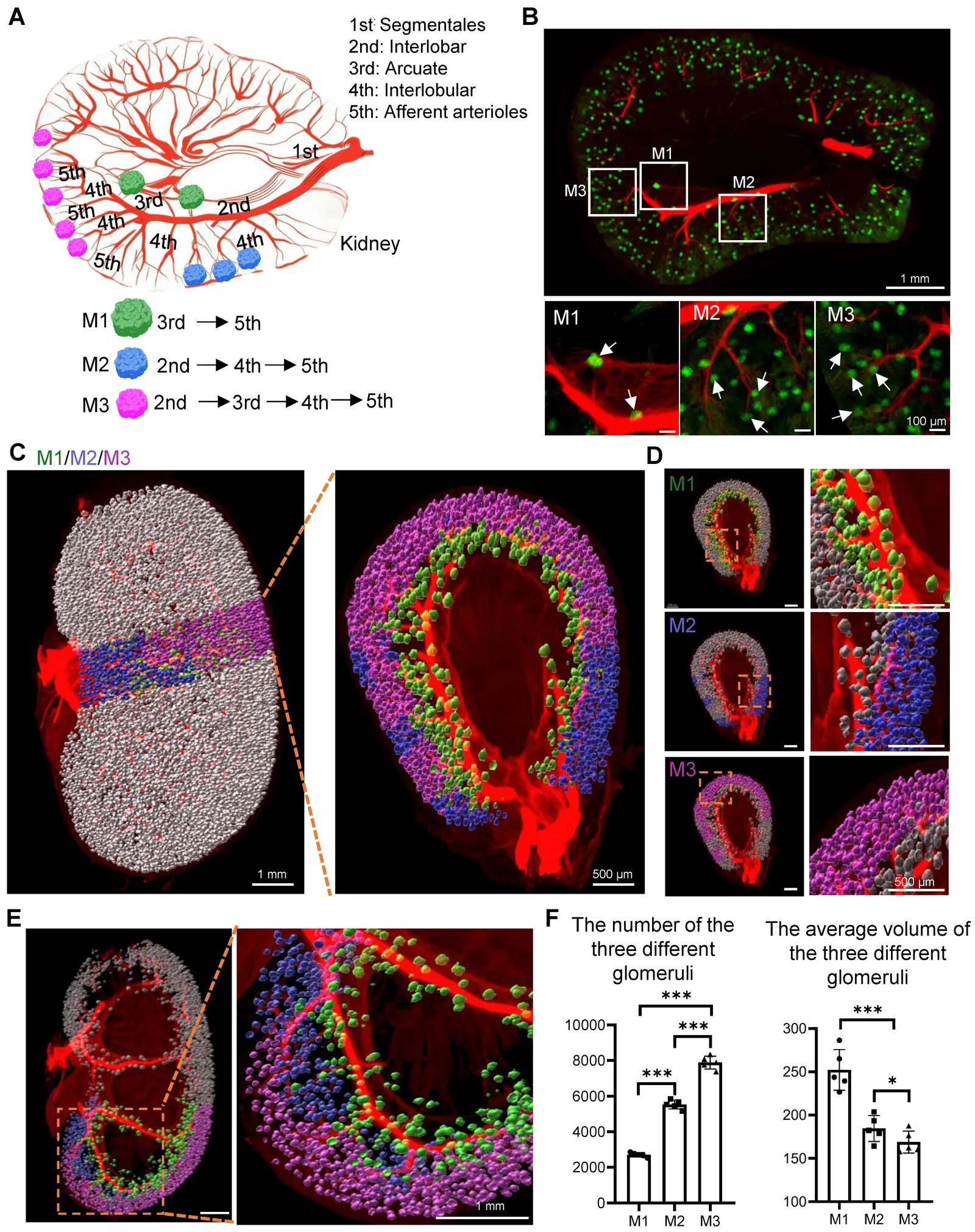
** The 3D visualization, classification, and precise analysis of glomeruli with distinct arterial origins in a normal mouse kidney. (A)** The schematic diagram of the classification of glomeruli origins (M1-M3). **(B)** Confocal microscopy results of kidney sections and magnified views of regions M1, M2, and M3. Green: glomeruli; Red: renal arteries, scale bar: 1 mm and 100 μm. **(C)** 3D reconstruction of three-type (M1-M3) glomeruli within a 1-mm-thick section of renal tissue located in the vertical mid-region of the kidney. Green: M1 glomeruli; Blue: M2 glomeruli; Red: M3 glomeruli, scale bar: 1 mm and 500 μm. **(D)** The 3D spatiotemporal distribution of M1-M3 glomeruli within the renal tissue in Figure 4C. Green: M1 glomeruli; Blue: M2 glomeruli; Red: M3 glomeruli, scale bar: 500 μm. **(E)** Longitudinal section of the kidney with classified glomerular origins from 3D reconstruction and its magnified partial view. Green: M1 glomeruli; Blue: M2 glomeruli; Red: M3 glomeruli, scale bar: 1 mm. **(F)** The quantitative statistical results for the number and mean volume of M1, M2, and M3 glomeruli, n = 5 (One kidney per mouse, 5 normal mice ).

Alterations in renal function are closely associated with changes in the spatial organization of renal microstructures 4. Because the spatial localization of glomeruli is closely associated with the origins of their afferent arterioles, quantitative analysis of complex 3D renal architecture—particularly glomerular origin—has remained challenging using conventional methods. However, EBDE clearing provided an effective approach to address this limitation. To better investigate the spatial heterogeneity of glomeruli, we classified them according to their origin, adopting the afferent arteriole classification method described by Donald J. Marsh et al. 31. As shown in Figure 4B, EBDE-based three-dimensional fluorescence imaging clearly revealed the hierarchical organization of renal arteries and glomeruli in coronal kidney sections (Figure 4B; white arrows indicate M1, M2, and M3 glomeruli). These findings demonstrated that EBDE clearing enabled three-dimensional tracking of spatial relationships between afferent arterioles and glomeruli throughout the kidney at micrometer-scale resolution, thereby allowing identification of three major glomerular subtypes based on arterial origin: M1, M2, and M3 (Figure 4A). M1 glomeruli originated from afferent arterioles arising from interlobar and arcuate arteries (3rd–5th). M2 glomeruli were supplied by afferent arterioles arising from relatively long interlobular arteries derived from interlobar arteries (2nd–4th–5th). M3 glomeruli originated from afferent arterioles derived from terminal interlobular arteries located at the distal end of the renal vascular tree (2nd-3rd-4th-5th) (Figure 4A, B, and Video S4). To characterize the distribution patterns of glomeruli with distinct afferent arteriolar origins, three-dimensional reconstruction and quantitative analyses of M1–M3 glomeruli were performed throughout the kidney using 1-mm-thick horizontal sections as analytical units (Figure 4C-E). Quantitative analyses revealed significant differences in the abundance of M1, M2, and M3 glomeruli, M1 glomeruli were the least numerous, while M3 glomeruli were the most abundant (Figure 4F). Additionally, Significant differences in glomerular volume were also observed among M1–M3 subtypes, with M1 glomeruli exhibiting the largest volume, followed by M2, whereas M3 glomeruli displayed the smallest size (Figure 4F). As revealed by the results, M1 glomeruli are predominantly distributed near the corticomedullary junction, whereas M2 glomeruli are mostly located in the cortical region adjacent to the renal pelvis. M3 glomeruli are the most abundant and widely distributed subtype. These three types of glomeruli together constitute the origin network of mouse glomerular afferent arterioles, and reflect the complexity and structural heterogeneity of glomerular distribution within the renal cortex, particularly their changes in the number and volume. This unique structural heterogeneity plays a significant role in hemodynamics and renal physiological function 36. In contrast to the study by Donald J. Marsh et al., which primarily summarized the functions and characteristics of afferent arterioles from different origins 31, we further differentiated and quantified glomeruli based on their sources. This approach provides deeper insights into the relationship between renal hemodynamics and physiological function.

### 3D visualization and quantitative analysis of renal arteries and glomeruli in UUO mice

Experimental unilateral ureteral obstruction (UUO) mouse is regarded as the standard model for studying renal tubulointerstitial fibrosis 37. In UUO mice, urinary tract obstruction leads to intrarenal hypertension, ischemia and hypoxia, inflammatory infiltration, and fibrosis, ultimately resulting in the constriction and stenosis of renal arterioles and the collapse of glomerular capillaries 38, 39. Glomerular count and renal arterial vessel density can directly quantify the extent of vascular injury, thereby objectively evaluating disease progression. However, current 3D whole-kidney imaging techniques, including micro-CT or super-resolution ultrasound, are unable to achieve precise and cross-scale 3D vascular imaging of the entire renal vasculature 40, 41. In this study, utilizing EBED clearing, we assessed the spatial structural relationships between the renal vasculature and the glomeruli.

**Figure 5:**
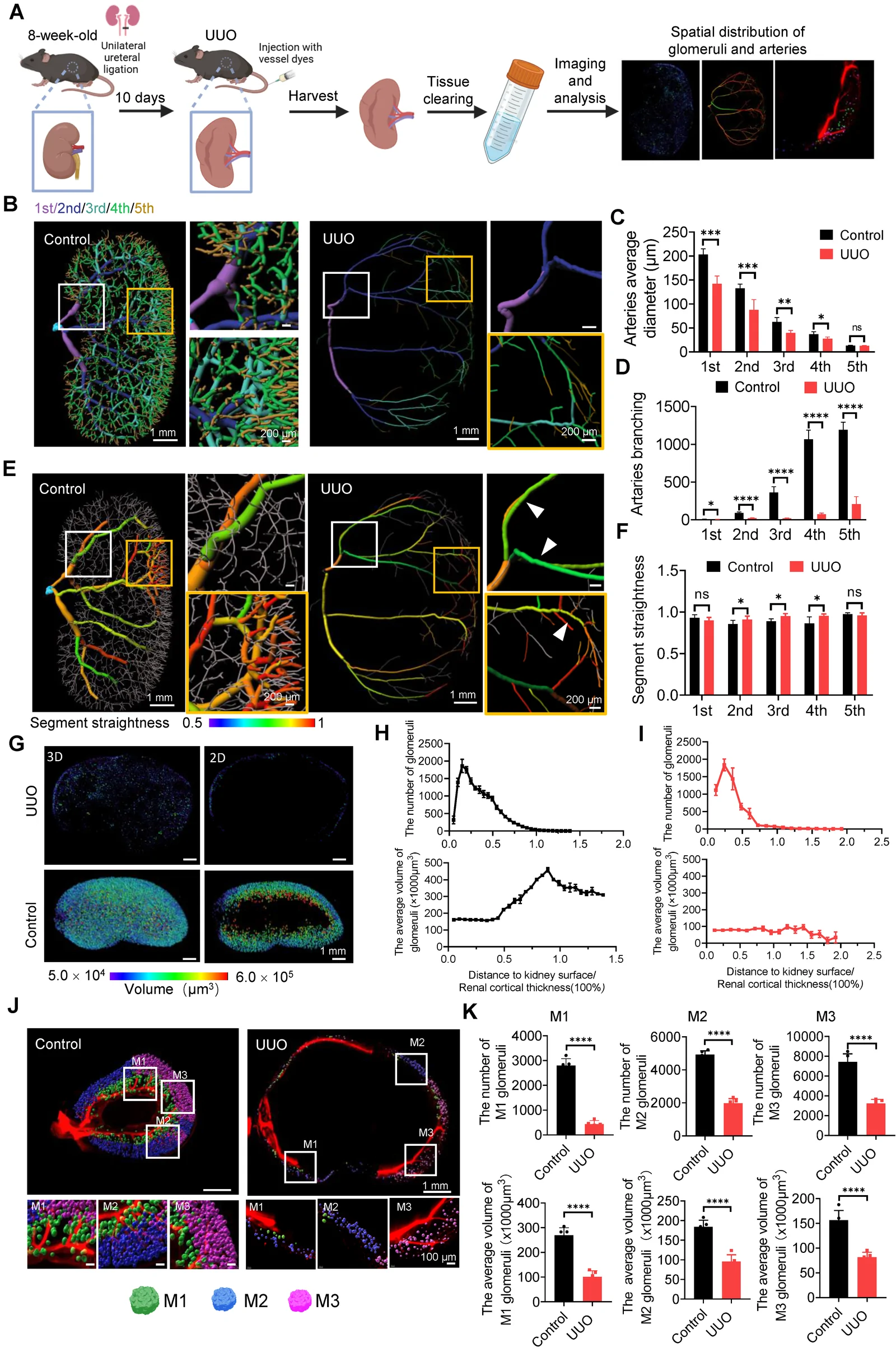
** 3D visualization and analysis of renal arteries and glomeruli in UUO mouse kidneys. (A)** The experimental workflow for 3D imaging of renal arteries and glomeruli in UUO mice. **(B)** The representative 3D reconstruction images of renal arterial diameter and branching patterns and magnified views in kidneys from control and UUO mice. Purple: 1st, segmentales; Blue: 2nd, interlobares; Light blue: 3rd, arcuate; Green: 4th, interlobular; Yellow: 5th, afferent arterioles, scale bar: 1 mm and 200 μm. (C, D) Quantitative analysis of average diameter, branch number of 1st-5th renal arteries from control and UUO mice, n = 6 (One kidney per mouse, 6 mice per group). **(E)** The representative images of arterial straightness and magnified views in kidneys from control and UUO mice, scale bar: 1 mm and 200 μm. **(F)** Quantitative analysis of straightness of 1st-5th renal arteries from control and UUO mice, n = 6 (One kidney per mouse, 6 mice per group). **(G)** The representative 3D reconstructed images of glomeruli in the whole kidney or the largest kidney cross-section (Thickness = 200 μm), scale bar: 1 mm. (H, I) The analysis of the correlation between glomerular number/volume and spatial location in normal **(H)** and UUO **(I)** mouse kidneys, n = 6 (One kidney per mouse, 6 mice per group). **(J)** Classification and magnified views of M1-M3 glomerular within a 1-mm-thick section of tissue located in the vertical mid-region of the kidney from control and UUO mice. Green: M1 glomeruli; Blue: M2 glomeruli; Red: M3 glomeruli, scale bar: 1 mm and 100 μm. **(K)** Quantitative analysis of the number and volume of M1-M3 glomeruli in the intact kidney from from control and UUO mice, n = 6 (One kidney per mouse, 6 mice per group). All values are presented as the mean ± SD. Statistical significance in **(C)**, **(D)**, **(F)**, and **(K)**, was assessed using an independent-sample t-test. *P<0.05, **P<0.01, ***P<0.001, ****P<0.0001, ns: no significance.

First, we used histopathological section staining to examine the kidneys of the two groups of mice and found that the number and volume of glomeruli per unit area in the kidneys of UUO mice were significantly reduced, accompanied by the proliferation of fibrous tissue in the renal interstitium and the destruction of kidney tissue structure (Figure S9). Furthermore, 3D imaging visualizations revealed that the kidneys of the UUO mice were markedly larger than those of the control mice. These results conclusively demonstrate the successful establishment of the UUO mouse model (Figure 5B, E, G, J, and Video S5, S6). To evaluate the feasibility of our labeling strategy for observing renal vascular in mice with UUO, we selected a high molecular weight dextran and EB as the kidney glomeruli and artery labeling, respectively, and performed 3D renderings of UUO and normal mouse kidneys (Figure 5A). The results shown that this method allowed us to simultaneously achieve high-resolution imaging of the glomeruli and arteries within the kidneys of normal and UUO mice (Figure 5B, E, and G).

To quantify renal vascular changes, we calculated renal arterial diameters and branches. As shown in Figure 5B, by using the EBED clearing, we achieved high-resolution 3D spatial imaging and reconstruction of arteries at different levels in the control and UUO kidneys. We observed a significant reduction in the diameters of segmental, interlobar, arcuate, and interlobular arteries (1st-4th) between UUO and control kidneys, and this reduction trend became more pronounced as vessel size increased. Whereas there were no statistically significant differences in the afferent arterioles (5th) (Figure 5C, Video S5). Besides, the number of vascular branches per level (1st-5th) in the kidneys of UUO group decreased sharply (Figure 5D, Video S5). The observed changes in arterial vasculature are consistent with previous micro-CT assessments of perfused murine kidneys 4. However, compared to micro-CT, our method detected a greater number of vascular branches and a narrower range of arterial diameter variations. This rapid and high-resolution detection method offers greater convenience and accuracy for the analysis of three-dimensional structures. The aforementioned pathological changes are likely attributable to hydronephrosis secondary to ureteral obstruction 38. Hydronephrosis exerts compressive force on the renal parenchyma and triggers significant vascular remodeling, which is particularly evident in larger vessels such as segmental arteries (1st), interlobar arteries (2nd), and arcuate arteries (3rd). Elevated intrarenal pressure, coupled with diminished blood flow, collectively induces renal ischemia and hypoxia, thereby promoting endothelial cell apoptosis, which in turn leads to destruction of the renal vascular structure and functional failure 42. The significant reduction in the diameters of arteries at all hierarchical levels and the number of arterial branches, reflects severe injury and destruction of the pre-glomerular renal vascular architecture. Based on relevant literature 38, 43, we hypothesize that the marked reduction in the diameters of large arteries (1st and 2nd) and small arteries (3rd and 4th) may be associated with mechanical compression and the hypoxic microenvironment, respectively.

Furthermore, we calculated the straightness of all arteries (1st-5th), and found that interlobar (2nd), arcuate (3rd) and interlobular arteries (4th) exhibited larger straightness in UUO mice, while other vessels (Segmentales, 1st; Afferent arterioles, 5th) showed no significant difference compared to the control group (Figure 5E, F, and Video S5). This may be attributed to the fact that segmental arteries are relatively fixed in position and thus less prone to mechanical twisting, and afferent arterioles typically exhibit severe constriction or rarefaction rather than tortuosity or deformation. Conversely, the pathological changes involving dynamic dilation and fibrosis within the UUO kidney render the interlobar, arcuate, and interlobular arteries traversing the renal cortex highly susceptible to morphological distortion 38. Unlike previous studies 4 that evaluated the straightness of individual vascular segments using micro-CT, our approach classifies vessels based on anatomical structures and calculates the straightness for each vascular order. This method provides a more profound analysis of the morphological alterations in the renal arterial vasculature under UUO pathological condition.

Besides, we reconstructed and quantified the glomeruli number and volume in UUO and control kidneys (Control) in 3D, and found that the number and average volume of glomeruli in the kidneys of UUO mice was significantly lower than that in the kidneys of control mice (Figure 5G, Figures S10A and S10B). The specific changes in the number of glomeruli with distance from the kidney surface in both groups were as follows: At approximately 100 μm from the kidney surface, the number of glomeruli in UUO mice initially showed a sharp increase followed by a steep decrease. Beyond the distance of 500 μm, there were almost no glomeruli presented in the kidney. In contrast, the inflection point at which the number of glomeruli transitions from increasing to decreasing is located at a distance of 200 μm from the kidney surface, and the depth at which glomeruli disappeared reached as far as 1000 μm (Figure S10C) in control kidney. To eliminate the confounding effects of pathological changes in kidney structure in diseased mice, we applied a correction when analyzing the correlations between glomerular number or mean volume and changes in spatial location (using the distance to kidney surface/Renal cortical thickness as the parameter for spatial positioning). The results showed that the spatial distribution patterns of glomerular numbers within the renal cortex were consistent between the two groups of mice, exhibiting a trend of initially increasing and then decreasing, with the peak value reached at the outer quarter of the renal cortex (Figure 5H and 5I). Furthermore, the average glomerular volume in UUO mice did not follow the same trend as that in the control group, specifically, they exhibited a trend of progressively decreasing size as the distance from the renal surface (Figure S10D) and the depth within the renal cortex increased (Figure 5H and 5I). Such findings are typically elusive in conventional two-dimensional studies. These results indicate that, by utilizing EBDE clearing technology, it is possible to successfully perform three-dimensional imaging and quantitative analysis of glomeruli in an acute UUO model. The kidney of UUO mice exhibited severe structural and functional renal impairment, manifested by enlarged kidney volume, cortical thinning, and structural distortion 38, 44. The significant reduction in glomerular number and volume may be attributed to the collapse and occlusion of capillary loops caused by intrarenal hypertension and hydronephrosis resulting from urinary obstruction 43.

To evaluate the relationship between glomeruli and arterial vessels, we quantitatively analyzed the number and volume of glomeruli from different origins. As shown in Figure 5J, the high-resolution spatial distribution of glomeruli and arteries in normal and UUO kidneys (Figure 5J and Video S6) was depicted. The statistical analysis revealed that the number and volume of M1, M2, and M3 glomeruli in the UUO kidneys were significantly reduced compared to those in the normal kidneys (Figure 5K). Among these, the reduction in the number of M1 glomeruli in the UUO kidneys was the most pronounced (decreasing nearly 5/6: 430 ± 154.1 vs 2797 ± 278.5), followed by M2 glomeruli (1984.4 ± 276.4 vs 4921 ± 234.8), and finally was M3 glomeruli (3240.2 ± 406.9 vs 7425.2 ± 823.8). Furthermore, the changes in the volume of each glomerular subtype mirrored the aforementioned trends observed in their numbers: M1 > M2 > M3 (M1, 101.6 ± 24.1 × 1000 μm^3^ vs 269.6 ± 30.2 × 1000 μm^3^; M2, 95.6 ± 17.5 × 1000 μm^3^ vs 184.0 ± 17.0 × 1000 μm^3^; M3, 81.7 ± 9.9 × 1000 μm^3^ vs 156.3 ± 19.7 × 1000 μm^3^) (Figure 5J, K, and Video S6). The reduced glomerular volume observed in this study is closely associated with glomerular atrophy and sclerosis, which aligns with the classic description by Chevalier et al 39, regarding glomerular sclerosis, tubular atrophy, and interstitial fibrosis following UUO. This reduction in glomerular volume may be attributed to insufficient upstream arterial supply, supporting the "preglomerular vascular injury hypothesis" 45, which posits that the loss or dysfunction of preglomerular vessels can lead to downstream ischemic atrophy of glomeruli. Through cross-scale three-dimensional vascular analysis, our study provides direct spatial and anatomical evidence for this hypothesis—a feat difficult to achieve with conventional two-dimensional sectioning techniques. However, the direction of this causality may be bidirectional: following glomerular sclerosis, afferent arterioles may undergo secondary occlusion (a "downstream-to-upstream" effect), rather than ischemia being driven by primary arterial lesions. Distinguishing between these two mechanisms requires dynamic observation or vascular perfusion experiments, which cannot be fully resolved by static three-dimensional imaging alone.

### 3D visualization and quantitative analysis of renal arteries and glomeruli in DN mice

**Figure 6:**
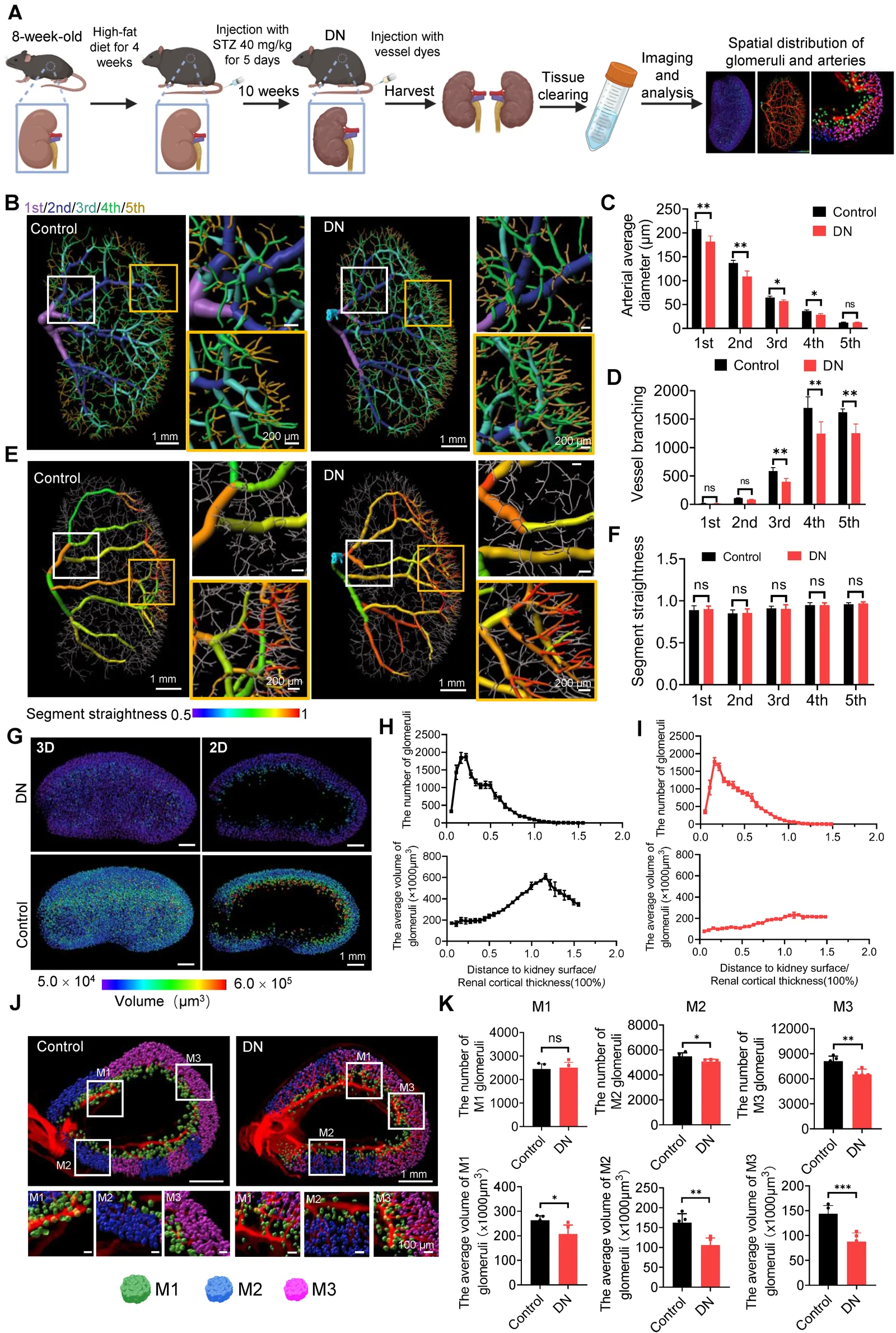
**3D visualization and analysis of the glomerular and arterial structures in DN mouse kidneys. (A)** The experimental workflow for 3D imaging of renal arteries and glomeruli in DN mice. **(B)** The representative 3D reconstruction images of renal arterial diameter and branching patterns and magnified views in kidneys from control and DN mice. Purple: 1st, segmentales; Blue: 2nd, interlobares; Light blue: 3rd, arcuate; Green: 4th, interlobular; Yellow: 5th, afferent arterioles, scale bar: 1 mm and 200 μm. (C, D) Quantitative analysis of average diameter, branch number of 1st-5th renal arteries from control and DN mice, n = 6 (One kidney per mouse, 6 mice per group). **(E)** The representative images of arterial straightness and magnified views in kidneys from control and DN mice, scale bar: 1 mm and 200 μm. **(F)** Quantitative analysis of straightness of 1st-5th renal arteries from control and DN mice, n = 6 (One kidney per mouse, 6 mice per group). **(G)** The representative 3D reconstructed images of glomeruli in the whole kidney or the largest kidney cross-section, scale bar: 1 mm. (H, I) The analysis of the correlation between glomerular number/volume and spatial location in normal **(H)** and DN **(I)** mouse kidneys, n = 6 (One kidney per mouse, 6 mice per group). **(J)** Classification and magnified views of M1-M3 glomerular within a 1-mm-thick section of tissue located in the vertical mid-region of the kidney from control and DN mice. Green: M1 glomeruli; Blue: M2 glomeruli; Red: M3 glomeruli, scale bar: 1 mm and 100 μm. **(K)** Quantitative analysis of the number and volume of M1-M3 glomeruli in the intact kidney from from control and DN mice, n = 6 (One kidney per mouse, 6 mice per group). All values are presented as the mean ± SD. Statistical significance in **(C)**, **(D)**, **(F)**, and **(K)**, was assessed using an independent-sample t-test. *P<0.05, **P<0.01, and ***P<0.001, ns: no significance.

Diabetic nephropathy (DN), characterized by progressive structural damage and functional decline of the kidneys, is the leading cause of end-stage renal disease (ESRD) 46 Among various diabetic models, streptozotocin (STZ)-induced diabetic mice are widely used to simulate type 2 diabetes and its renal complications, primarily due to their stable pathological features, high reproducibility, and close similarity to human DN progression. STZ selectively damages pancreatic β-cells, leading to insulin deficiency, persistent hyperglycemia, and subsequent renal injury—including glomerular sclerosis, microvascular rarefaction, and arterial remodeling. Notably, most previous studies on DN have focused on microvascular lesions (e.g., peritubular capillaries, glomerular capillaries) 47, 48 while the pathological changes of macro-vessels (especially the renal arterial tree) and their spatial correlation with glomerular damage have been largely overlooked. Therefore, the systematically exploration of spatiotemporal changes in the renal arteries and glomeruli of STZ-induced diabetic mice using 3D visualization and quantitative analysis is crucial for elucidating the mechanisms underlying the comprehensive renal vascular damage and enriching its pathological research in DN. Currently, the pathological research involving the 3D visualization of renal vasculature and glomerular in DN remains a complete void. Therefore, we established a DN mouse by administering a high-fat diet in combination with intraperitoneal injections of STZ. Subsequent histopathological examination of the renal tissues from these model mice confirmed the successful establishment of the DN model, and revealed characteristic pathological changes, including glomerular atrophy, reduced glomerular count, and renal fibrosis (Figure S11). Finally, utilizing EBDE clearing technology, we visualized the spatial distribution of renal arteries and glomeruli within the kidneys of the DN mice ((Figure 6A).

Firstly, the statistical analysis of the arteries, we performed 3D reconstruction and quantitative analysis of the entire renal vasculature in DN mice (Figure 6B). The measurements revealed significant reductions in segmental (1st), interlobar (2nd), arcuate (3rd), and interlobular arterial (4th) diameters in DN mice, while afferent arteriolar (5th) diameter remained unchanged compared to controls (Figure 6C). Vessel branching counts in 3D and local magnifications are shown in Figure 6D, and revealed that there were significant reductions in arcuate (3rd), interlobular (4th), and arcuate (5th) arteries. The segmental (1st) and interlobar (2nd) vessel branching showed no significant changes (Figure 6E). As shown in Figure 6F, the analysis of arterial straightness at various levels in the two groups of kidneys revealed that there were no significant differences. Our three-dimensional quantitative analysis reveals that arterial remodeling in diabetic nephropathy (DN) exhibits a unique proximal-to-distal gradient pattern: the diameters of segmental and interlobar arteries (1st and 2nd) are significantly reduced; arcuate and interlobular arteries (3rd and 4th) simultaneously show decreased diameters and branch loss; whereas the diameter of afferent arterioles (5th) remains unchanged. This finding contrasts sharply with the pattern observed in obstructive nephropathy, where deep cortical glomeruli (M1) are the most vulnerable, suggesting that the vascular pathology in DN is disease-specific 49, 50. Specifically, the stenosis of proximal large arteries may represent a compensatory response aimed at protecting glomeruli from high-pressure stress 51, while the loss of branches in arcuate and interlobular arteries marks a turning point from compensation to decompensation. Compared to micro-CT 4, which relies on intravascular perfusion and is prone to filling artifacts in fibrotic tissues, and to SHANEL 25 using dextran tracing, which struggles to distinguish arteries from veins and cannot differentiate functional vasoconstriction from structural stenosis, our structural labeling method provides definitive evidence through direct visualization of the vessel wall. This clearly demonstrates that the diameter reduction represents true vascular wall remodeling rather than inadequate perfusion. Nevertheless, it is possible that the proximal arterial narrowing is partially attributable to vascular wall edema induced by hyperosmolarity rather than structural loss 50. Similarly, the apparent reduction in branching could be an artifact of incomplete labeling caused by AGEs deposition impeding dye penetration 52. Future studies should employ longitudinal time-series analyses to determine the temporal sequence of vascular injury across different hierarchical levels, combined with *in vivo* hemodynamic assessments to validate the correlation between structural alterations and functional changes.

To identify pathological changes in glomeruli showed that there was no significant difference in the number between the DN and the control group, and the spatial distribution trends of glomerular number in both were consistent, whether based on the normalizing distance (Distance to kidney surface/Renal cortical thickness) or the distance from the kidney surface (Figures 6H, 6I and Figure S12A,). However, DN mice exhibited a slight reduction in the number of glomeruli at various depths within 500 μm from the kidney surface (Figure S12C). Subsequently, we compared the spatial variation of glomerular average volume between the two groups and found that their spatial distribution trends were consistent. However, the mean glomerular volume was lower in the DN group relative to controls (Figure 6G, Figure S12B). When comparing glomeruli at matching anatomical positions, glomerular volume was substantially reduced in DN mice. Moreover, the disparity between groups became more pronounced deeper into the renal cortex and at greater distances from the renal capsule (Figure 6I, Figure S12D). Collectively, these volumetric reductions indicate that STZ-induced diabetes drives progressive glomerular atrophy along the cortical depth axis.

Next, we analyzed the spatiotemporal distribution of different glomeruli (M1-M3) within the kidneys of DN mice. The results indicated that, except for M1 glomeruli, the number and volume of glomeruli across all other grades within the kidneys of DN mice exhibited varying degrees of reduction. However, this downward trend was not as pronounced as that in UUO mice (Figure 6J). Quantitative analysis revealed that, compared to the control group, the number of M2 (5061 ± 202.3 vs 5486.4 ± 285.2) and M3 (6501 ± 661.6 vs 8113.2 ± 603) glomeruli decreased in the DN group, with the reduction in M3 glomeruli being more pronounced. While the number of M1(2498.2 ± 230.3 vs 2443.2 ± 222.2) glomeruli remained largely unchanged. Furthermore, the volumes of M1 (207.4 ± 36.4 × 1000 μm^3^ vs 263 ± 18.5× 1000 μm^3^), M2 (105.5 ± 18.4 × 1000 μm^3^ vs 162.5 ± 22.3 × 1000 μm^3^), and M3 (87.8 ± 17.3 × 1000 μm^3^ vs 143.5 ± 16.8 × 1000 μm^3^) glomeruli showed a significant reduction, and the volume in M3 glomeruli was decreased the most pronounced (Figure 6K). This observation implies that high blood flow and elevated filtration pressure are more likely to trigger glomerular hypertension and podocyte injury, subsequently resulting in atrophy 53. In the DN mice, M2 and M3 glomeruli are predominantly concentrated in the superficial renal cortex, where alterations in their number and volume might be linked to inadequate superficial renal blood flow and oxygenation 13. Conversely, M1 glomeruli, located closer to the medulla, exhibited only a slight reduction in volume, likely due to relatively better blood perfusion in renal medulla. The reduction in the number of superficial glomeruli (M3) corresponds precisely to the significant loss of branches in interlobular arteries (4th). Given that M3 glomeruli are situated at the most distal ends of the arterial tree, they are the most sensitive to upstream branch loss. Conversely, the lack of significant reduction in deep glomeruli (M1) aligns with the maintained diameter of afferent arterioles (5th), further supporting the hypothesis that vascular supply determines glomerular vulnerability 52. However, the decrease in superficial glomeruli may also be attributed to non-vascular factors. Finally, our findings highlight the critical role and potential of 3D vascular spatial analysis model in revealing localized glomerular damage in DN.

## Conclusion

In summary, we established EBDE clearing, an integrated three-dimensional (3D) imaging workflow that enables simultaneous high-resolution visualization and quantitative analysis of renal arteries and glomeruli throughout intact kidneys. By combining Evans blue-mediated arterial labeling, dextran-based glomerular labeling, and Ethanol-ECi tissue clearing, this strategy achieved comprehensive visualization of the renal arterial tree and glomerular architecture across multiple hierarchical levels. Using this approach, we systematically characterized renal vascular and glomerular remodeling in unilateral ureteral obstruction (UUO) and diabetic nephropathy (DN) mouse models. Quantitative analyses revealed distinct pathological patterns between the two disease models, with UUO exhibiting more pronounced alterations in renal arterial architecture and glomerular organization, whereas DN primarily displayed relatively mild cortical glomerular remodeling under the current experimental conditions. Furthermore, our classification framework based on glomerular arterial origin (M1–M3) enabled refined spatial characterization of glomerular heterogeneity in normal and diseased kidneys. Collectively, these findings not only provide new insights into renal vascular and glomerular remodeling during disease progression but also establish EBDE clearing as a promising platform for whole-organ 3D visualization and quantitative assessment of renal microarchitecture. This strategy may facilitate future investigations of renal pathophysiology and could potentially be extended to vascular imaging in other organs.

## Supplementary Material

Supplementary figures and tables, movie legends.

Supplementary movie 1.

Supplementary movie 2.

Supplementary movie 3.

Supplementary movie 4.

Supplementary movie 5.

Supplementary movie 6.

Supplementary movie 7.

Supplementary movie 8.

## Data Availability

All data from this study are available upon request with the consent of the corresponding author.

## References

[1] Daniel E, Azizoglu DB, Ryan AR, Walji TA, Chaney CP, Sutton GI (2018). Spatiotemporal heterogeneity and patterning of developing renal blood vessels. Angiogenesis.

[2] Molema G, Aird WC (2012). Vascular heterogeneity in the kidney. Semin Nephrol.

[3] Chade AR (2013). Renal vascular structure and rarefaction. Compr Physiol.

[4] Knuechel R, Ehling J, Bábíčková J, Gremse F, Lammers T, Boor P (2016). Quantitative micro-computed tomography imaging of vascular dysfunction in progressive kidney diseases. J Am Soc Nephrol.

[5] Li Y, Cao J, Zhang Q, Li J, Li X, Zhou H (2024). Precise reconstruction of the entire mouse kidney at cellular resolution. Biomed Opt Express.

[6] Cravedi P, Remuzzi G (2013). Pathophysiology of proteinuria and its value as an outcome measure in chronic kidney disease. Br J Clin Pharmacol.

[7] Romagnani P, Tang SCW, Weins A, Huber TB, Osafo C, Anders H-J (2025). Podocytopathies. Nat Rev Dis Primers.

[8] Funes Hernandez M, Bhalla V, Isom RT (2022). Hypothesis: Accessory renal arteries may be an overlooked cause of renin-dependent hypertension. J Hum Hypertens.

[9] Deferrari G, Cipriani A, La Porta E (2021). Renal dysfunction in cardiovascular diseases and its consequences. J Nephrol.

[10] Laffin LJ, Bakris GL (2021). Intersection between chronic kidney disease and cardiovascular disease. Curr Cardiol Rep.

[11] Klinkhammer BM, Lammers T, Mottaghy FM, Kiessling F, Floege J, Boor P (2021). Non-invasive molecular imaging of kidney diseases. Nat Rev Nephrol.

[12] Caroli A, Remuzzi A, Lerman LO (2021). Basic principles and new advances in kidney imaging. Kidney Int.

[13] Zhao S, Zhang X, Bailey K, Pai S, Zhao Y, Chen YS (2025). Label-free dual-modal photoacoustic/ultrasound localization imaging for studying acute kidney injury. Adv Sci (Weinh).

[14] Chen Q, Yu J, Rush BM, Stocker SD, Tan RJ, Kim K (2020). Ultrasound super-resolution imaging provides a noninvasive assessment of renal microvasculature changes during mouse acute kidney injury. Kidney Int.

[15] Hlushchuk R, Zubler C, Barré S, Correa Shokiche C, Schaad L, Röthlisberger R (2017). Cutting-edge microangio-CT: new dimensions in vascular imaging and kidney morphometry. Am J Physiol Renal Physiol.

[16] Baldelomar EJ, Charlton JR, deRonde KA, Bennett KM (2019). *In vivo* measurements of kidney glomerular number and size in healthy and Os/+ mice using MRI. Am J Physiol Renal Physiol.

[17] Kurniawan ND, Amar AJ, Cullen-McEwen LA, Combes AN, Hayatudin R, Kassianos AJ (2025). Counting glomeruli in human kidney specimens using ex vivo MRI without contrast agents. Magn Reson Med.

[18] Heilmann M, Neudecker S, Wolf I, Gubhaju L, Sticht C, Schock-Kusch D (2012). Quantification of glomerular number and size distribution in normal rat kidneys using magnetic resonance imaging. Nephrol Dial Transplant.

[19] Fumoto S, Nishimura K, Nishida K, Kawakami S (2016). Three-dimensional imaging of the intracellular fate of plasmid DNA and transgene expression: ZsGreen1 and tissue clearing method CUBIC are an optimal combination for multicolor deep imaging in murine tissues. PLoS ONE.

[20] Sakamoto DM, Tamura I, Yi B, Hasegawa S, Saito Y, Yamada N (2024). Whole-body and whole-organ 3D imaging of hypoxia using an activatable covalent fluorescent probe compatible with tissue clearing. ACS nano.

[21] Puelles VG, Combes AN, Bertram JF (2021). Clearly imaging and quantifying the kidney in 3D. Kidney Int.

[22] Klingberg A, Hasenberg A, Ludwig-Portugall I, Medyukhina A, Männ L, Brenzel A (2017). Fully automated evaluation of total glomerular number and capillary tuft size in nephritic kidneys using lightsheet microscopy. J Am Soc Nephrol.

[23] Østergaard MV, Sembach FE, Skytte JL, Roostalu U, Secher T, Overgaard A (2020). Automated lmage analyses of glomerular hypertrophy in a mouse model of diabetic nephropathy. Kidney360.

[24] Zhu J, Liu X, Xu J, Deng Y, Wang P, Liu Z (2023). A versatile vessel casting method for fine mapping of vascular networks using a hydrogel-based lipophilic dye solution. Cell Rep Methods.

[25] Zhao S, Todorov MI, Cai R, Maskari RA, Steinke H, Kemter E (2020). Cellular and molecular probing of intact human organs. Cell.

[26] Bai L, Wu Y, Dai W, Shi Q, Wu L, Zhang J (2024). An efficient and fast method for labeling and analyzing mouse glomeruli. JoVE.

[27] Chevalier RL, Forbes MS, Thornhill BA (2009). Ureteral obstruction as a model of renal interstitial fibrosis and obstructive nephropathy. Kidney Int.

[28] Xie Z, Wang X, Luo X, Yan J, Zhang J, Sun R (2023). Activated AMPK mitigates diabetes-related cognitive dysfunction by inhibiting hippocampal ferroptosis. Biochem Pharmacol.

[29] Hasegawa S, Susaki EA, Tanaka T, Komaba H, Wada T, Fukagawa M (2019). Comprehensive three-dimensional analysis (CUBIC-kidney) visualizes abnormal renal sympathetic nerves after ischemia/reperfusion injury. Kidney Int.

[30] Qi Y, Yu T, Xu J, Wan P, Ma Y, Zhu J (2019). FDISCO: Advanced solvent-based clearing method for imaging whole organs. Sci Adv.

[31] Marsh DJ, Postnov DD, Rowland DJ, Wexler AS, Sosnovtseva OV, Holstein-Rathlou N-H (2017). Architecture of the rat nephron-arterial network: analysis with micro-computed tomography. Am J Physiol Renal Physiol.

[32] Honeycutt SE, N'Guetta P-EY, Hardesty DM, Xiong Y, Cooper SL, Stevenson MJ (2023). Netrin 1 directs vascular patterning and maturity in the developing kidney. Development.

[33] Heinle H, Lindner V (1984). The binding of Evans blue to collagen and elastin in elastic tissue. Arch Int Physiol Biochim.

[34] Weizsäcker HW, Zierler E, Juch H (2014). A simple method for vital staining of elastin in arterial tissue. Biomed Tech (Berl).

[35] Chaweewannakorn C, Harada T, Nyasha MR, Koide M, Shikama Y, Hagiwara Y (2021). Imaging of muscle activity-induced morphometric changes in fibril network of myofascia by two-photon microscopy. J Anat.

[36] Nordsletten DA, Blackett S, Bentley MD, Ritman EL, Smith NP (2006). Structural morphology of renal vasculature. Am J Physiol Heart Circ Physiol.

[37] Wilson DR, Wilson DDR (1980). Pathophysiology of obstructive nephropathy. Kidney Int.

[38] Nørregaard R, Mutsaers HAM, Frøkiær J, Kwon T-H (2023). Obstructive nephropathy and molecular pathophysiology of renal interstitial fibrosis. Physiol Rev.

[39] Chevalier RL (1999). Molecular and cellular pathophysiology of obstructive nephropathy. Pediatr Nephrol.

[40] Oh D, Lee D, Heo J, Kweon J, Yong U, Jang J (2023). Contrast agent-free 3D renal ultrafast doppler imaging reveals vascular dysfunction in acute and diabetic kidney diseases. Adv Sci (Weinh).

[41] Foiret J, Zhang H, Ilovitsh T, Mahakian L, Tam S, Ferrara KW (2017). Ultrasound localization microscopy to image and assess microvasculature in a rat kidney. Sci Rep.

[42] Wang J-H, Lin Y-L, Hsu B-G (2025). Endothelial dysfunction in chronic kidney disease: Mechanisms, biomarkers, diagnostics, and therapeutic strategies. Tzu Chi Med J.

[43] Klahr S, Morrison A, Buerkert J (1980). Effects of urinary tract obstruction on renal function. Contrib Nephrol.

[44] Klahr S, Morrissey J (2002). Obstructive nephropathy and renal fibrosis. Am J Physiol Renal Physiol.

[45] Khater Y, Barakat N, Shokeir A, Samy A, Karrouf G (2025). Renal fibrosis progression following partial unilateral ureteral obstruction: mechanisms and therapeutic insights. World J Urol.

[46] Kamli-Salino SEJ, Brown PAJ, Haschler TN, Liang L, Feliers D, Wilson HM (2023). Induction of experimental diabetes and diabetic nephropathy using anomer-equilibrated streptozotocin in male C57Bl/6J mice. Biochem Biophys Res Commun.

[47] Steegh FMEG, Keijbeck AA, de Hoogt PA, Rademakers T, Houben AJHM, Reesink KD (2024). Capillary rarefaction: a missing link in renal and cardiovascular disease?. Angiogenesis.

[48] Afsar B, Afsar RE, Dagel T, Kaya E, Erus S, Ortiz A (2018). Capillary rarefaction from the kidney point of view. Clinical kidney journal.

[49] Mkhize S (2024). Histopathological variations in diabetic nephropathy. Int J Pathol Sci.

[50] Shi J, Yang Y, Cheng A, Xu G, He F (2020). Metabolism of vascular smooth muscle cells in vascular diseases. Am J Physiol Heart Circ Physiol.

[51] Wani ZA, Ahmed S, Saleh A, Anna VR, Fahelelbom KM, Raju SK (2025). Biomarkers in diabetic nephropathy: A comprehensive review of their role in early detection and disease progression monitoring. Diabetes Res Clin Pract.

[52] Qasim H, Dibian S, Hayajneh A, Khattab K, Leoni MLG, Varrassi G (2025). Histopathology of diabetic nephropathy: Beyond glomerular basement membrane thickening. Cureus.

[53] Zhang H, Huang L, Yang Y, Qiu L, He Q, Liu J (2023). Evaluation of early diabetic kidney disease using Ultrasound localization microscopy: A feasibility study. J of Ultrasound Medicine.

