# Supplementary material for: EBDE clearing: A strategy for simultaneous 3D visualization and quantitative analysis of both renal arteries and glomeruli
Source: Theranostics. 2026 Aug 24;16(15):8987–9006. doi: 10.7150/thno.138951 (PMC13549429; doi:10.7150/thno.138951)
Supplement: Supplementary file 1 [file thnov16p8987s1.pdf]

Supplementary materials

EBDE clearing: A strategy for simultaneous 3D visualization and quantitative analysis of both renal arteries and glomeruli

Yaping Wu[1], Qian Shen[2], Yan Liang[1], Lin Bai[1], Jinsai Wu[3], Qiuxiao Shi[1], Lei Wu[1], Menqi Wang[4]*, Yin Liu[5]*, and Kui Huang[6,7,8]*

[1]Core Facilities of West China Hospital, Sichuan University, Chengdu 610041, Sichuan, China

[2]Department of Radiology, The Affiliated Stomatological Hospital, Southwest Medical University, Luzhou 646002, China.

[3]Laboratory of Mitochondrial Metabolism and Perioperative Medicine, National-Local Joint Engineering Research Centre of Translational Medicine of Anesthesiology, West China Hospital of Sichuan University, Chengdu 610041, Sichuan, China.

[4]Department of Neurosurgery, West China Hospital, Sichuan University, Chengdu 610041, Sichuan, China.

[5]Department of Laboratory Medicine, West China Hospital, Sichuan University, Chengdu 610041, China.

[6]State Key Laboratory of Oral Diseases National Clinical Research Center for Oral Diseases, West China Hospital of Stomatology, Sichuan University, Chengdu 610041, China

[7]Luzhou Key Laboratory of Oral & Maxillofacial Reconstruction and Regeneration, Luzhou 646600, China

[8]Department of Oral and Maxillofacial Surgery, The Affiliated Stomatological Hospital, Southwest Medical University, Luzhou 646002, China.

*Corresponding author: Menqi Wang, Yin Liu, and Kui Huang, E-mail: wmq_1101@scu.edu.cn, liuyin14@wchscu.edu.cn, huangkui@swmu.edu.cn.

Supplementary figures

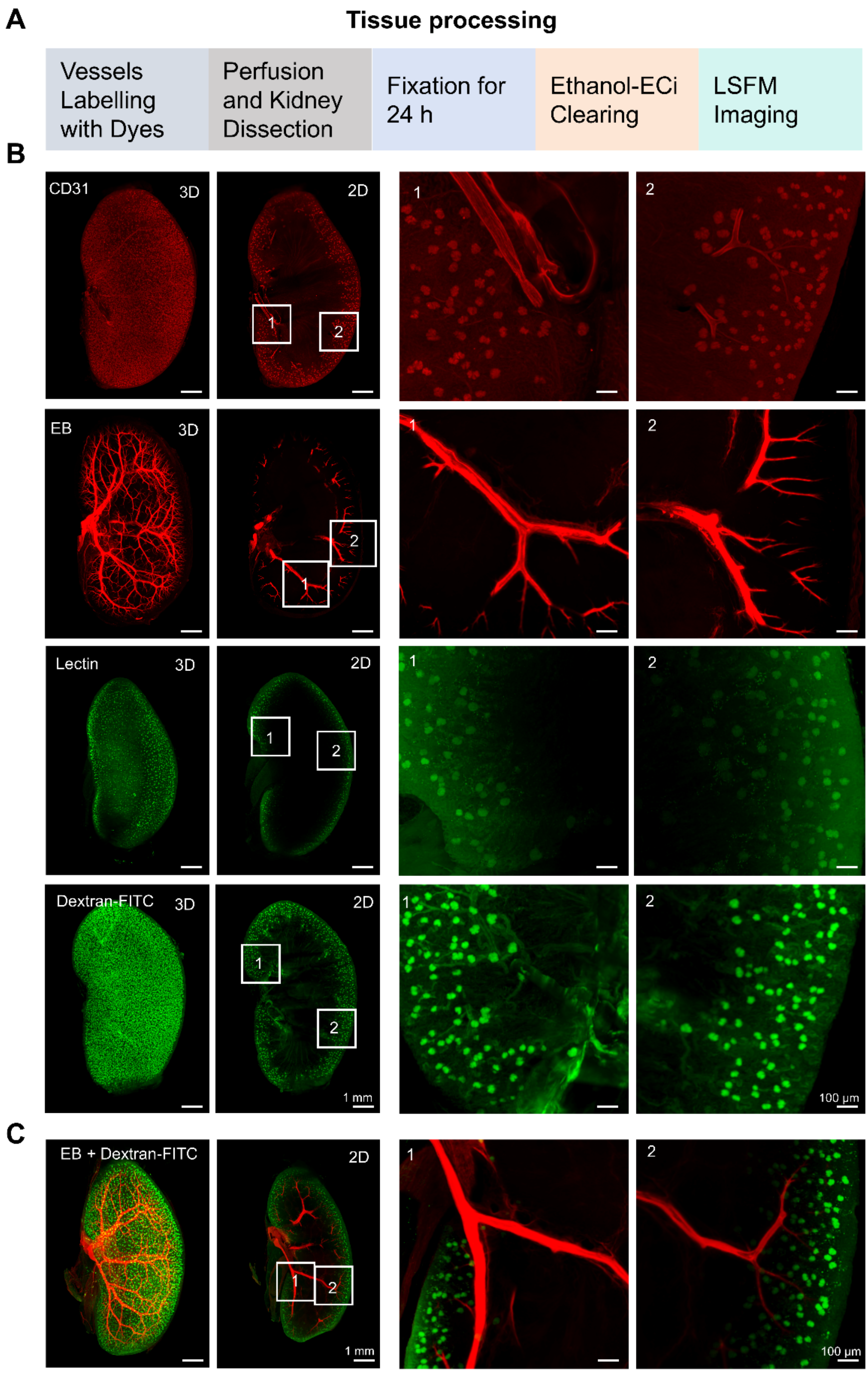

Figure S1. The comparison of the efficacy of different vascular dyes in labeling mouse

renal arteries and glomeruli. (A) Experimental workflow for renal arteries and glomeruli labeling and their three-dimensional imaging. (B) Representative images of 3D reconstructions and 2D cross-sections of renal arteries and glomeruli within 200 μm-thick normal kidney tissue, labeled with CD31, EB, lectin, and dextran. (C) Representative images of 3D reconstructions and 2D cross-sections of renal arteries and glomeruli within 200 μm-thick normal kidney tissue, labeled with EB and dextran. Scale bar: 1 mm and 100 μm.

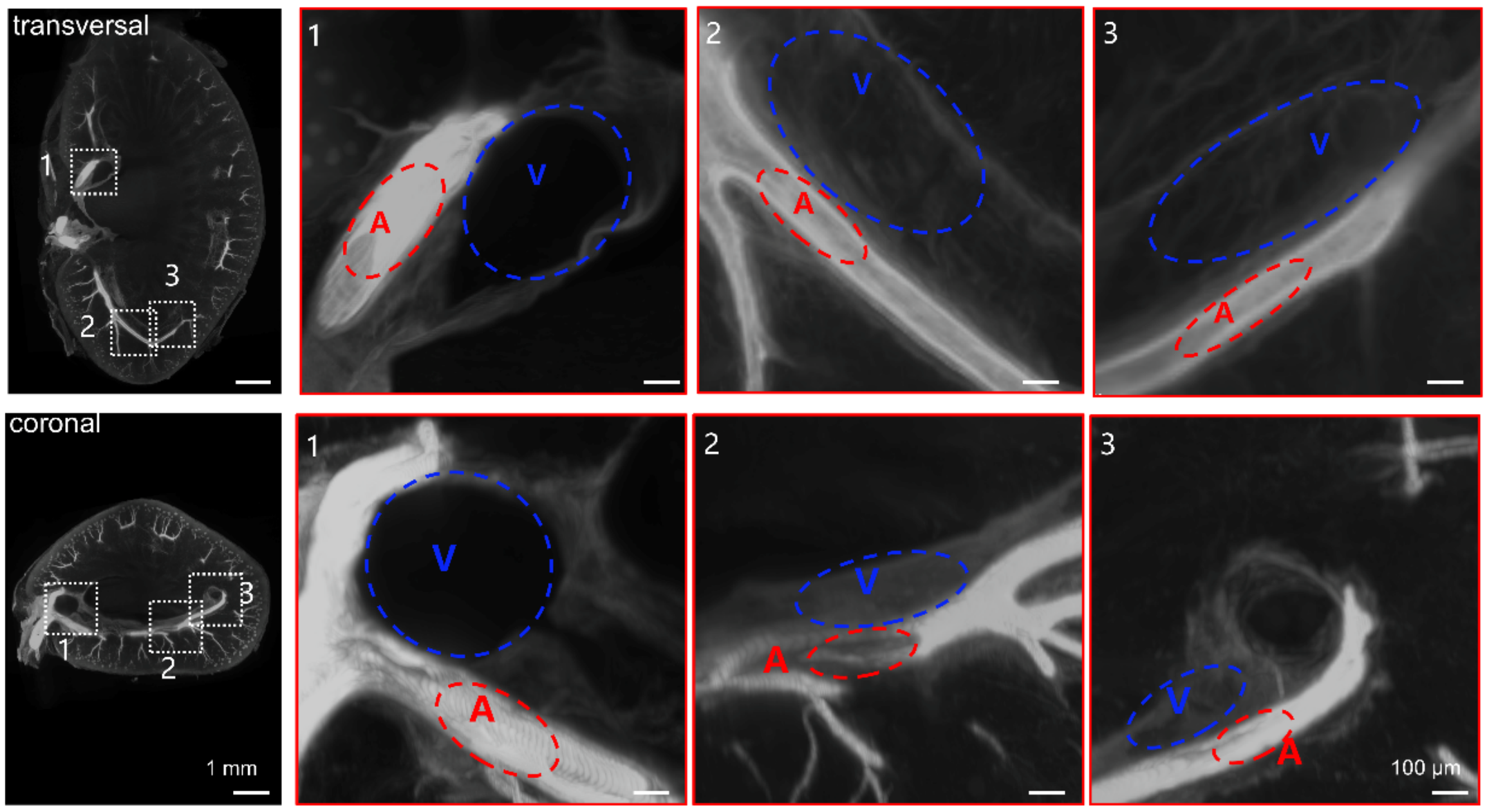

Figure S2. EB enables effective labeling of renal arteries at different levels. A: artery, V: vein. White arrows: glomeruli, scale bar: 1mm and 100 μm.

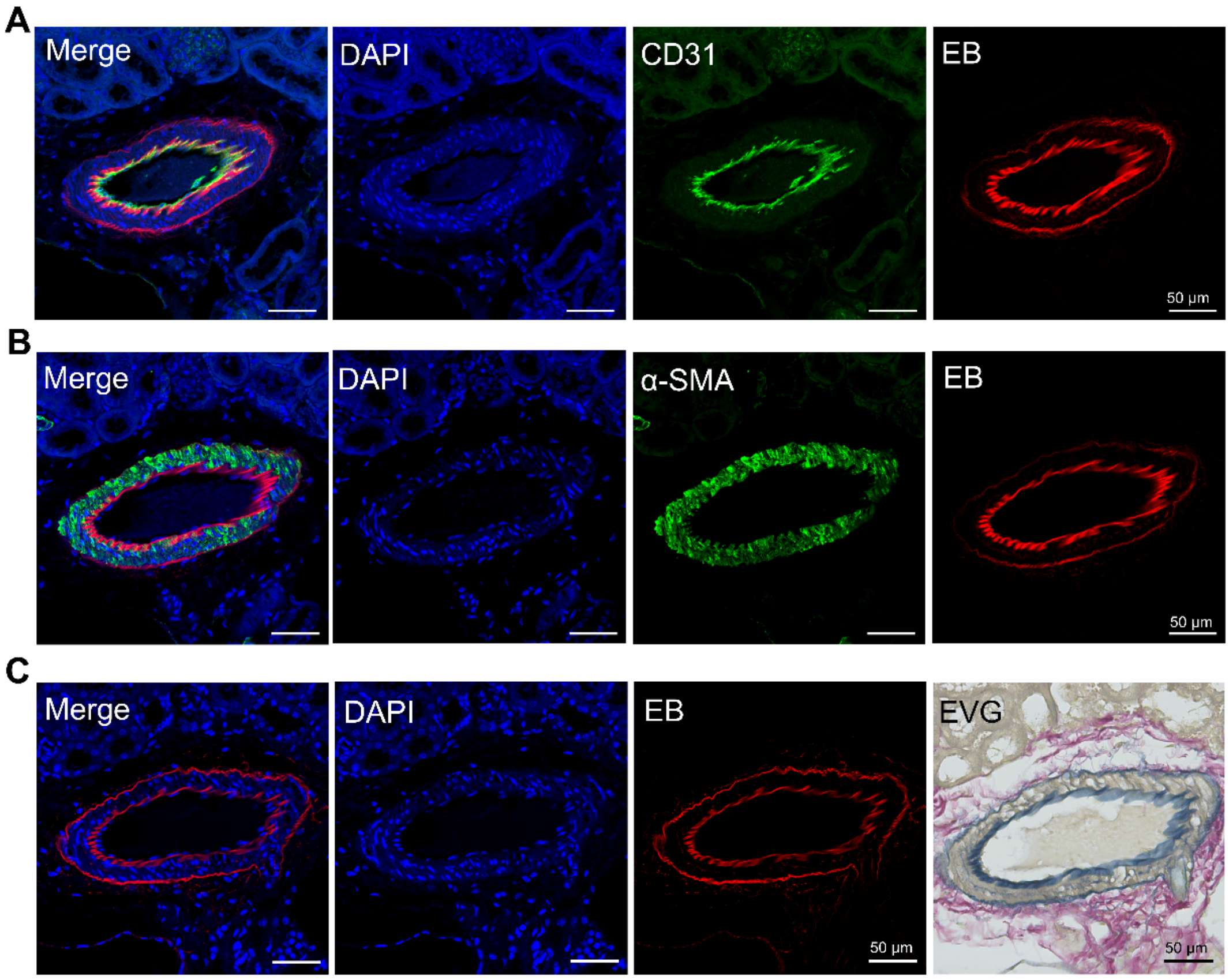

Figure S3. EB can specifically bind to elastic fibers in arteries. (A, B) Immunofluorescence images of CD31 and α-SMA staining in paraffin sections of normal mouse kidney. (C) EB immunofluorescence and EVG staining images of the same paraffin-embedded mouse kidney section. Scale bar: 50 μm.

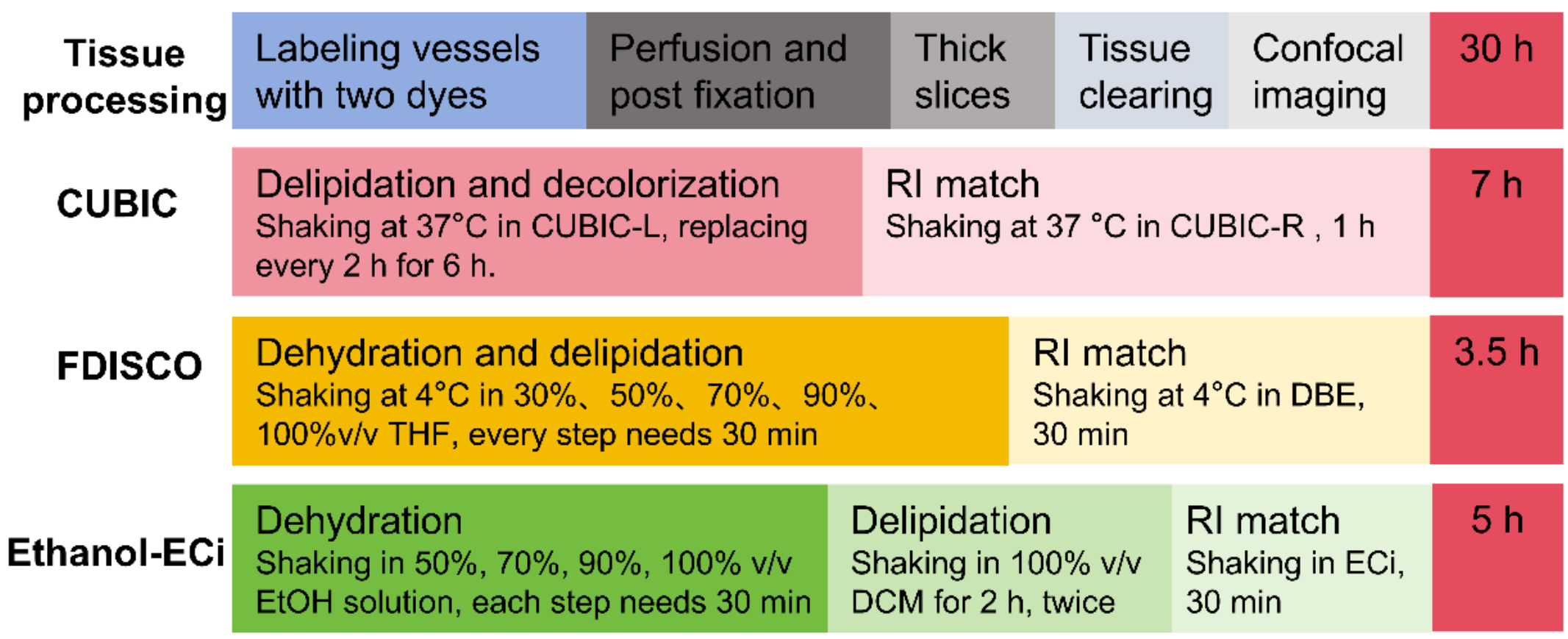

Figure S4. The experimental workflow of different clearing methods used in this study.

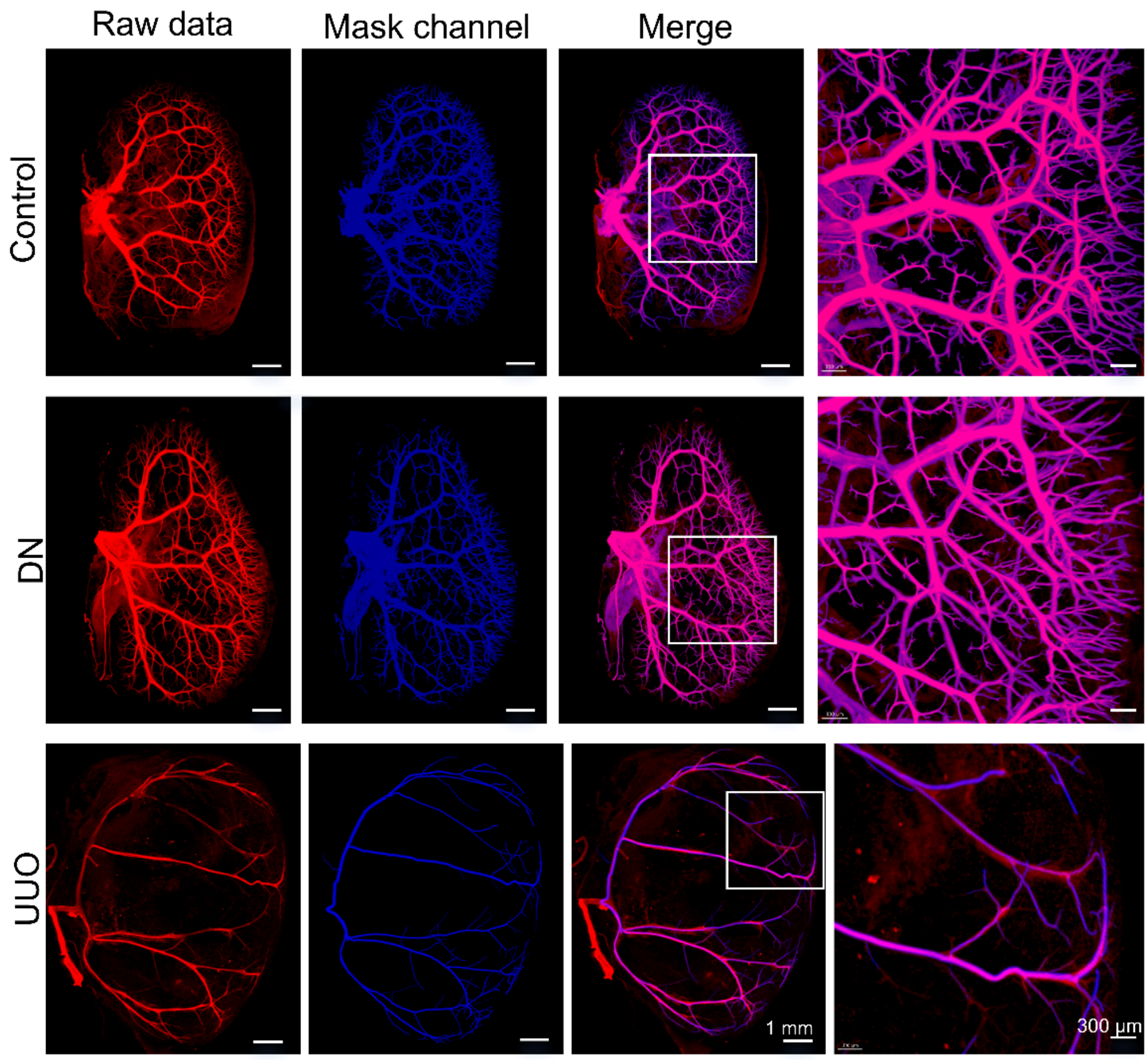

Figure S5. The 3D fluorescence imaging, automatically reconstructed images, and magnified views of arteries in the kidneys of Control, DN, and UUO mice following EBDE clearing. Scale bar: 300 μm, and 1 mm.

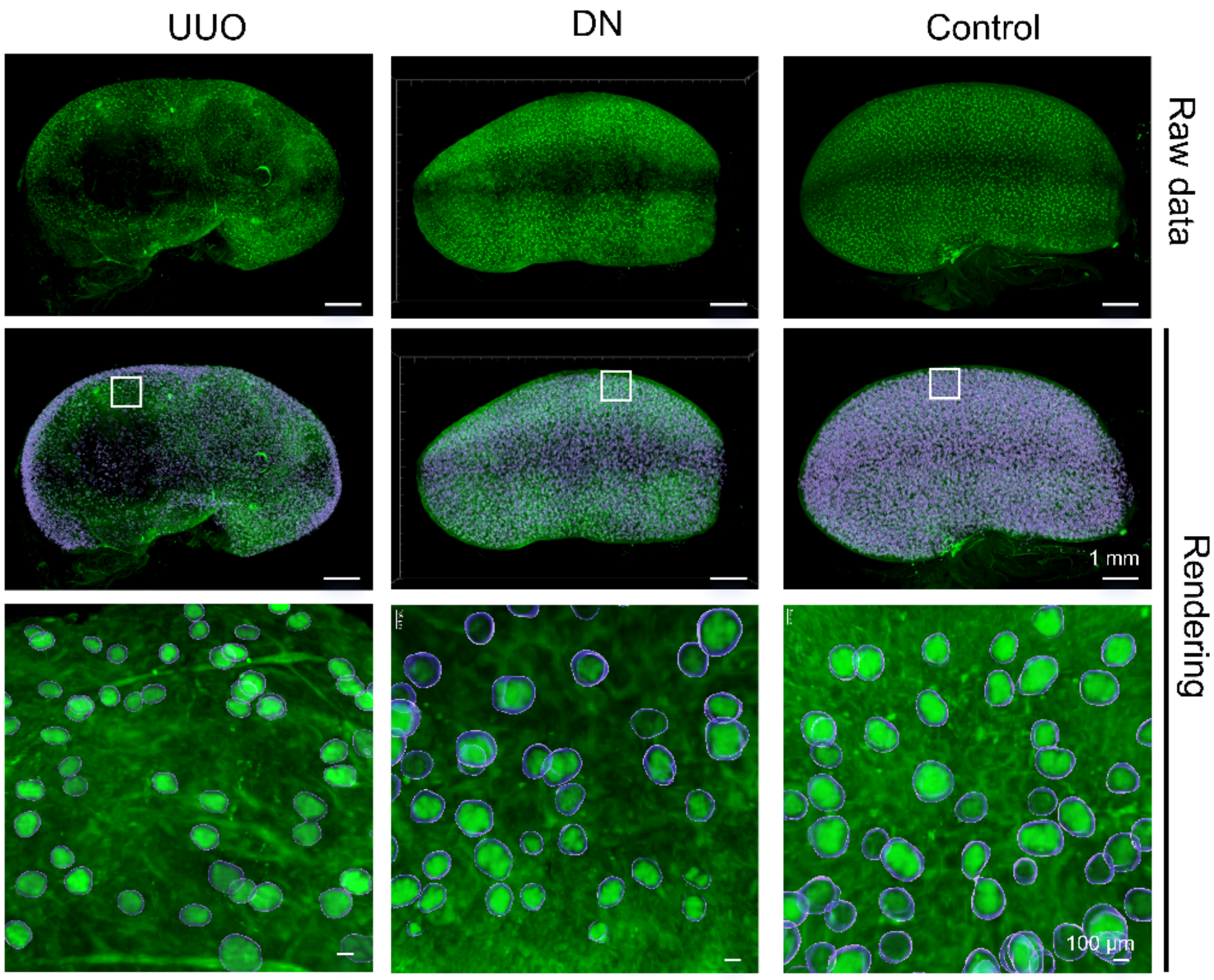

Figure S6. The 3D fluorescence imaging, automatically reconstructed images, and magnified views of glomeruli in the kidneys of Control, DN, and UUO mice following EBDE clearing. Scale bar: 100 μm, and 1 mm.

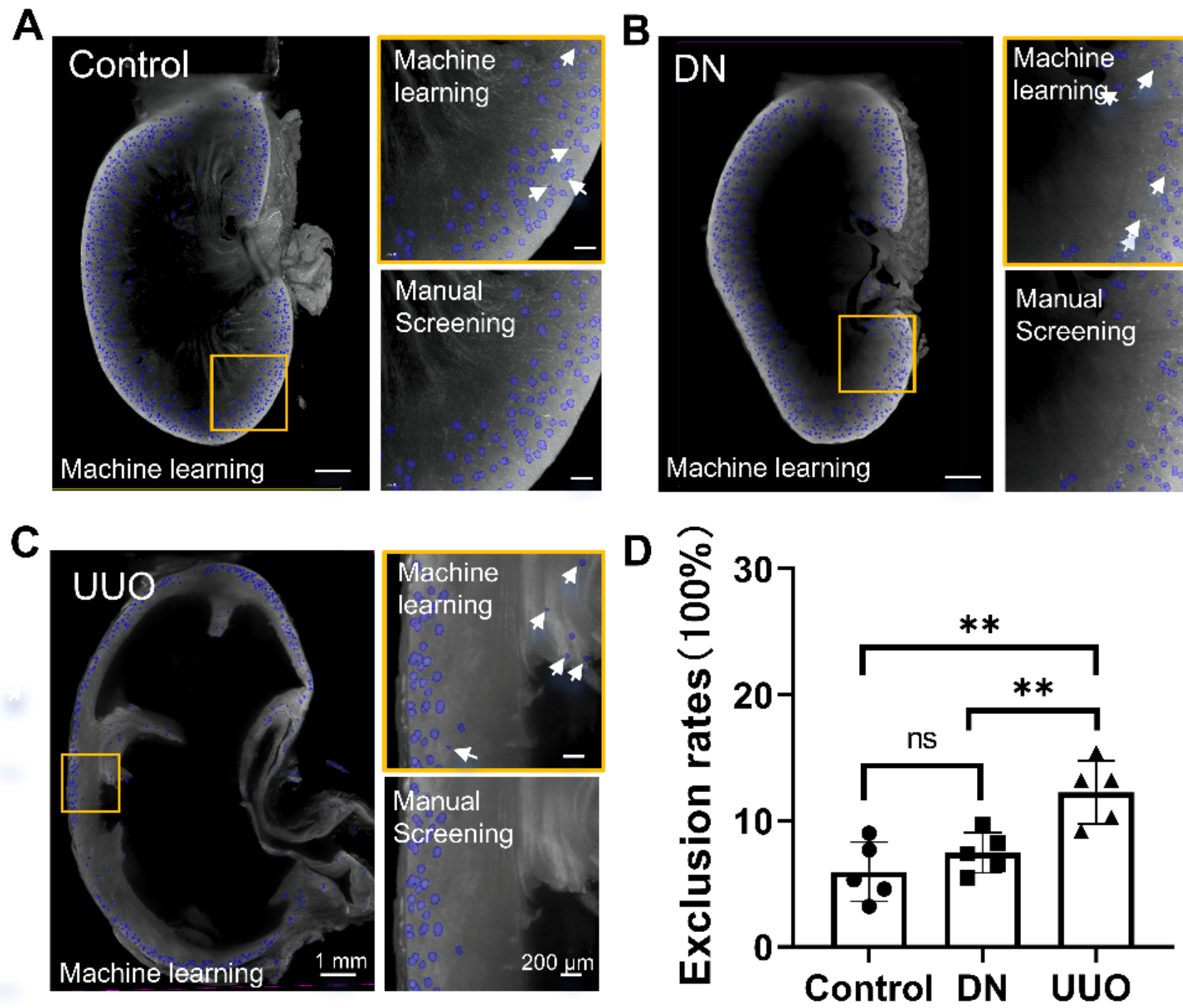

Figure S7. The manual exclusion of non-glomerular signals in normal or diseased

kidneys. (A) The automated 3D reconstruction of glomeruli using machine learning and manual exclusion of non-glomerular signals in normal kidneys. (B) The automated 3D reconstruction of glomeruli using machine learning and manual exclusion of non-glomerular signals in DN kidneys. (C) The automated 3D reconstruction of glomeruli using machine learning and manual exclusion of non-glomerular signals in UUO kidneys. (D) Quantitative analysis of manual exclusion rates, n = 5. Scale bar: 200 μm and 1 mm.

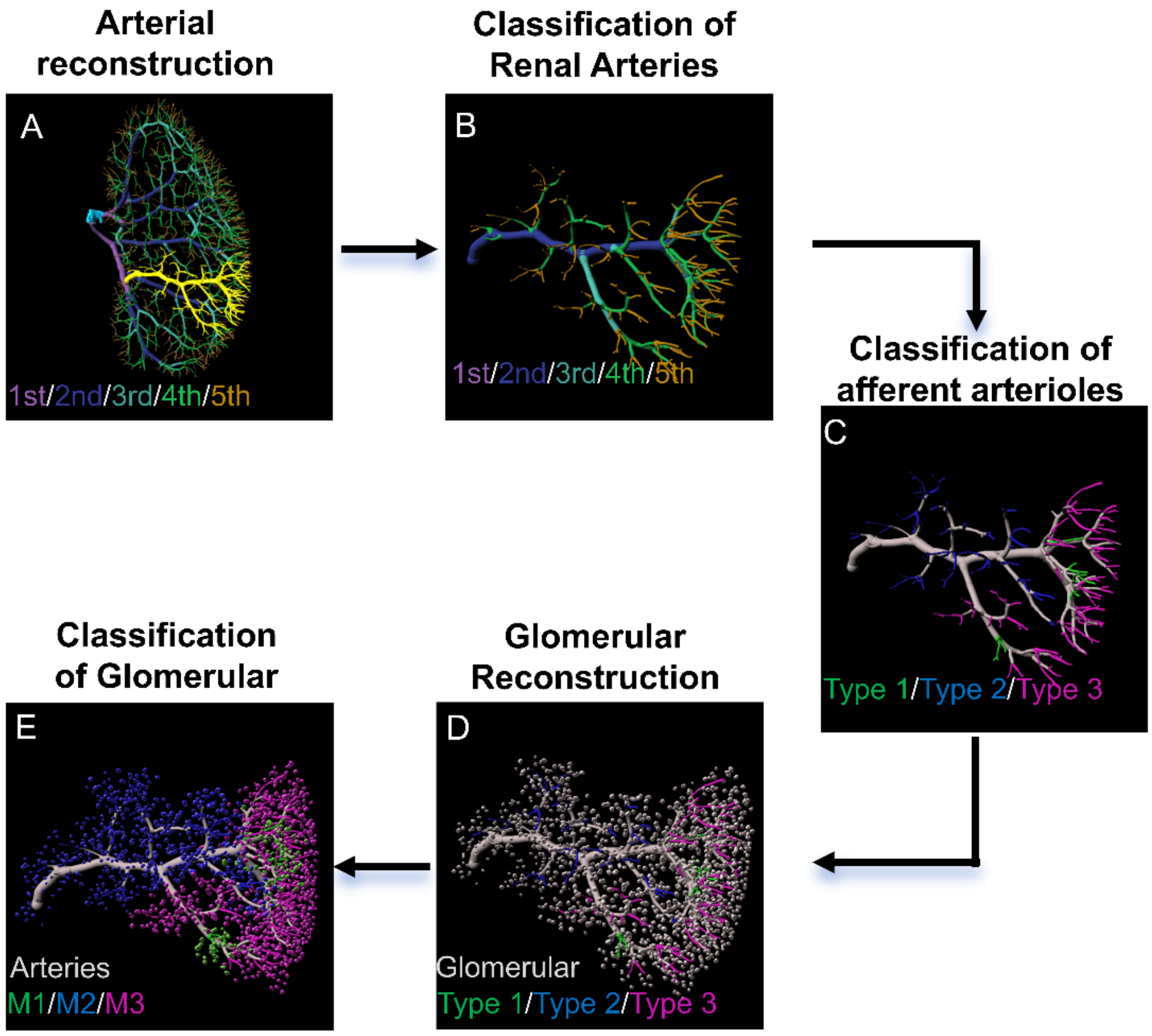

Figure S8. The flowchart for M1–M3 glomerular classification. (A) Three-dimensional reconstruction of renal arteries in normal mice. Purple: 1st, Segmentales; Blue: 2nd, Interlobares; Light blue: 3rd, Arcuate; Green: 4th, Interlobular; Yellow: 5th, Afferent arterioles. (B) Classification of renal arteries in normal mice. Purple: 1st, Segmentales; Blue: 2nd, Interlobares; Light blue: 3rd, Arcuate; Green: 4th, Interlobular; Yellow: 5th, Afferent arterioles. (C) Classification of afferent arterioles in normal mice. Green: Type 1 afferent arterioles; Blue: Type 2 afferent arterioles; Red: Type 3 afferent arterioles. (D) Three-dimensional reconstruction of glomeruli in normal mice. Gray: Glomeruli; Green: Type 1 Afferent arterioles; Blue: Type 2 Afferent arterioles; Red: Type 3 Afferent arterioles. (E) Classification of glomeruli in normal mice. Green: M1 Glomeruli; Blue: M2 Glomeruli; Red: M3 Glomeruli.

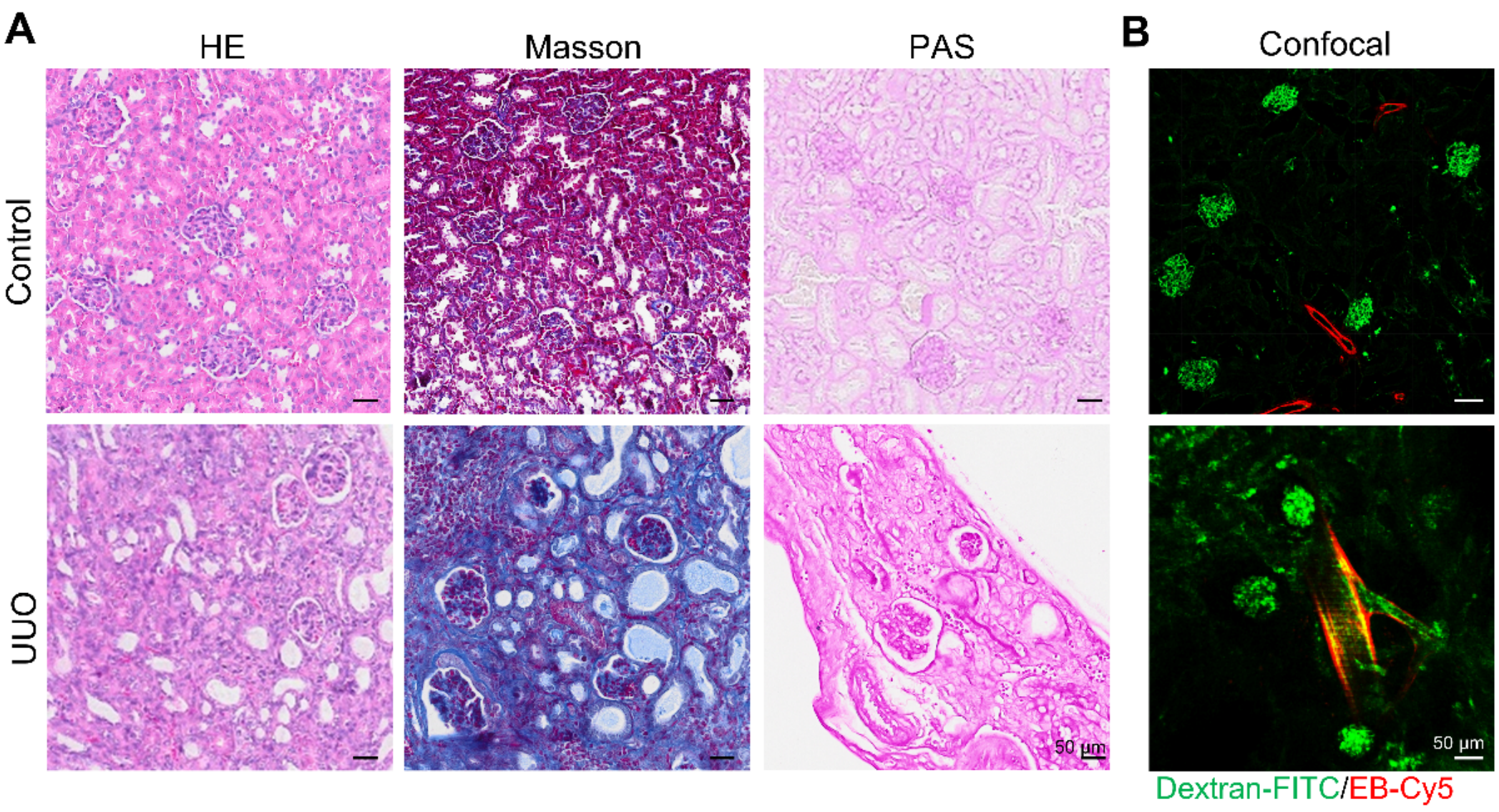

Figure S9. The pathological staining images of kidneys from Control and UUO mice. (A) The representative images of the HE, Masson, and PAS staining of kidney sections from UUO and Control groups. (B). The representative confocal fluorescence images of kidney from normal and UUO mice labeled with Dextran-FITC/EB-Cy5. Green: glomerulus, Red: renal arteries, scale bar: 50 μm.

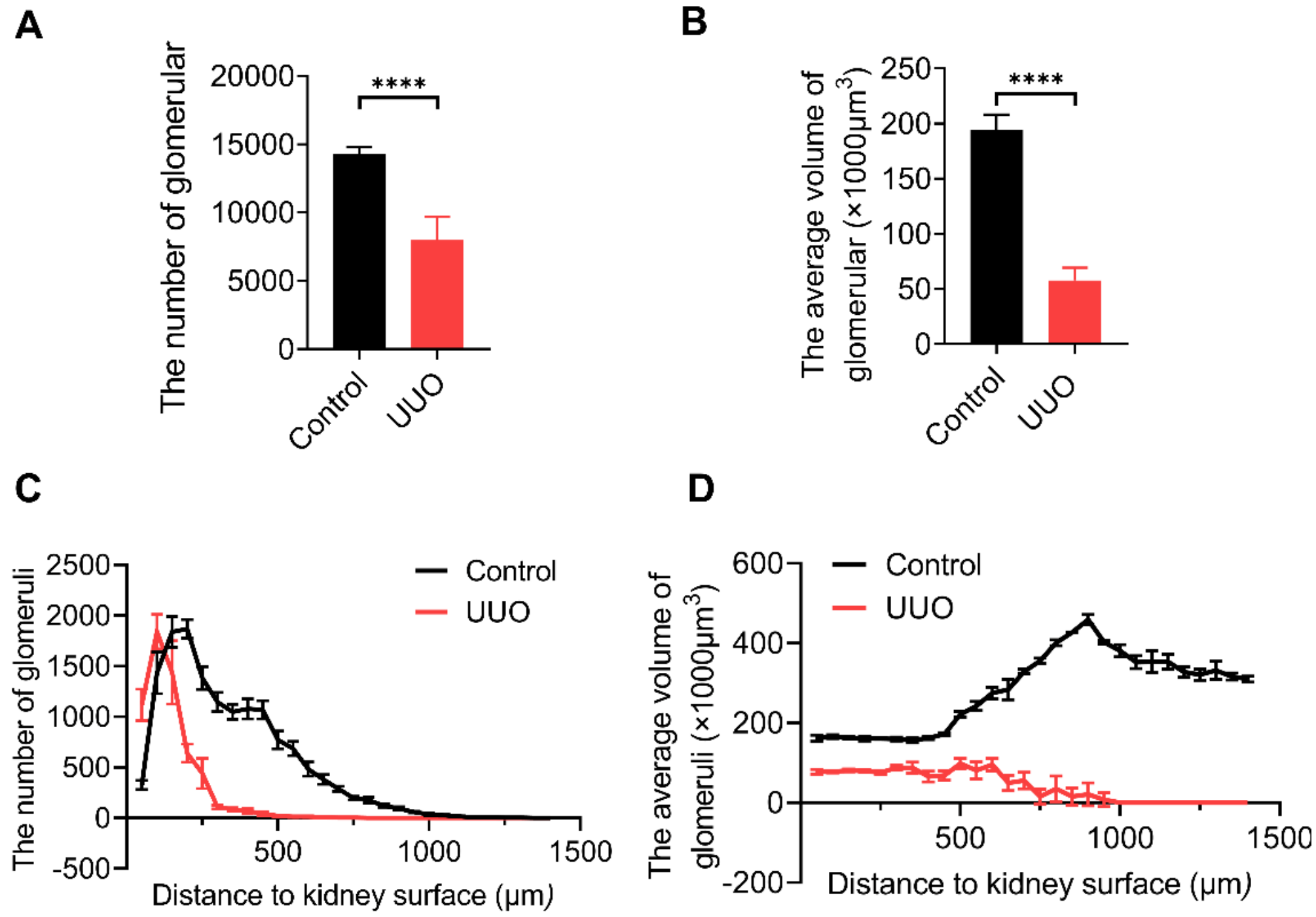

Figure S10. The quantitatively analyze the number (A) and average volume (B) of glomeruli in the Control and UUO mice, n = 6 (One kidney per mouse, 6 mice per

group). The quantitative determination of the relationships between glomerular number (C), average volume (D), and their specific locations within the kidney of Control and UUO mice, n = 6. All values are presented as the mean ± SD. Statistical significance in (A, B) was assessed using an independent-sample t-test. ***P<0.001.

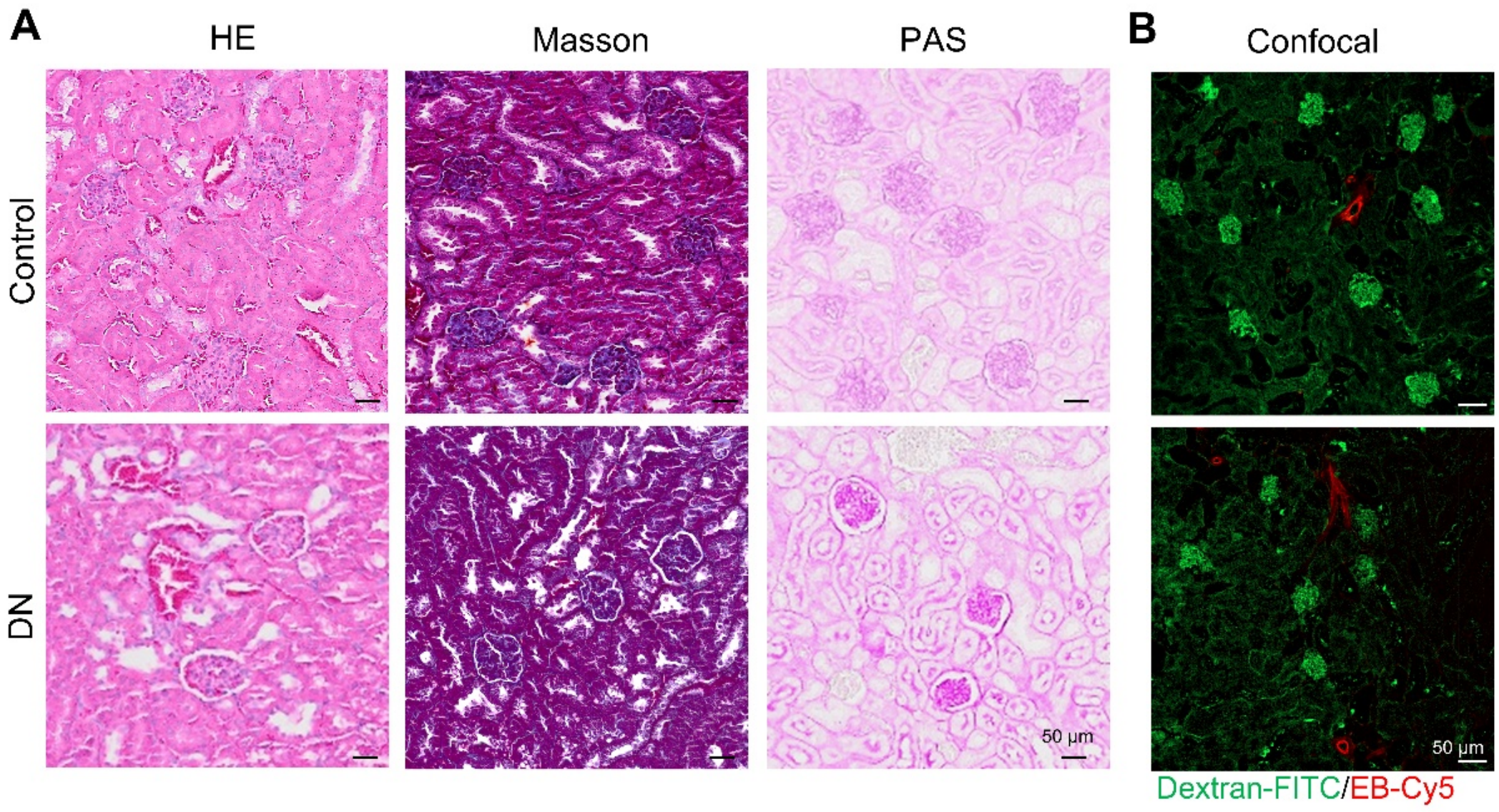

Figure S11. The pathological staining images of kidneys from Control and DN mice. (A) The representative images of the HE, Masson, and PAS staining of kidney sections from Control and DN groups. (B) The representative confocal fluorescence images of kidney from normal and DN mice labeled with Dextran-FITC/EB-Cy5. Green: glomerulus, Red: renal arteries, scale bar: 50 µm.

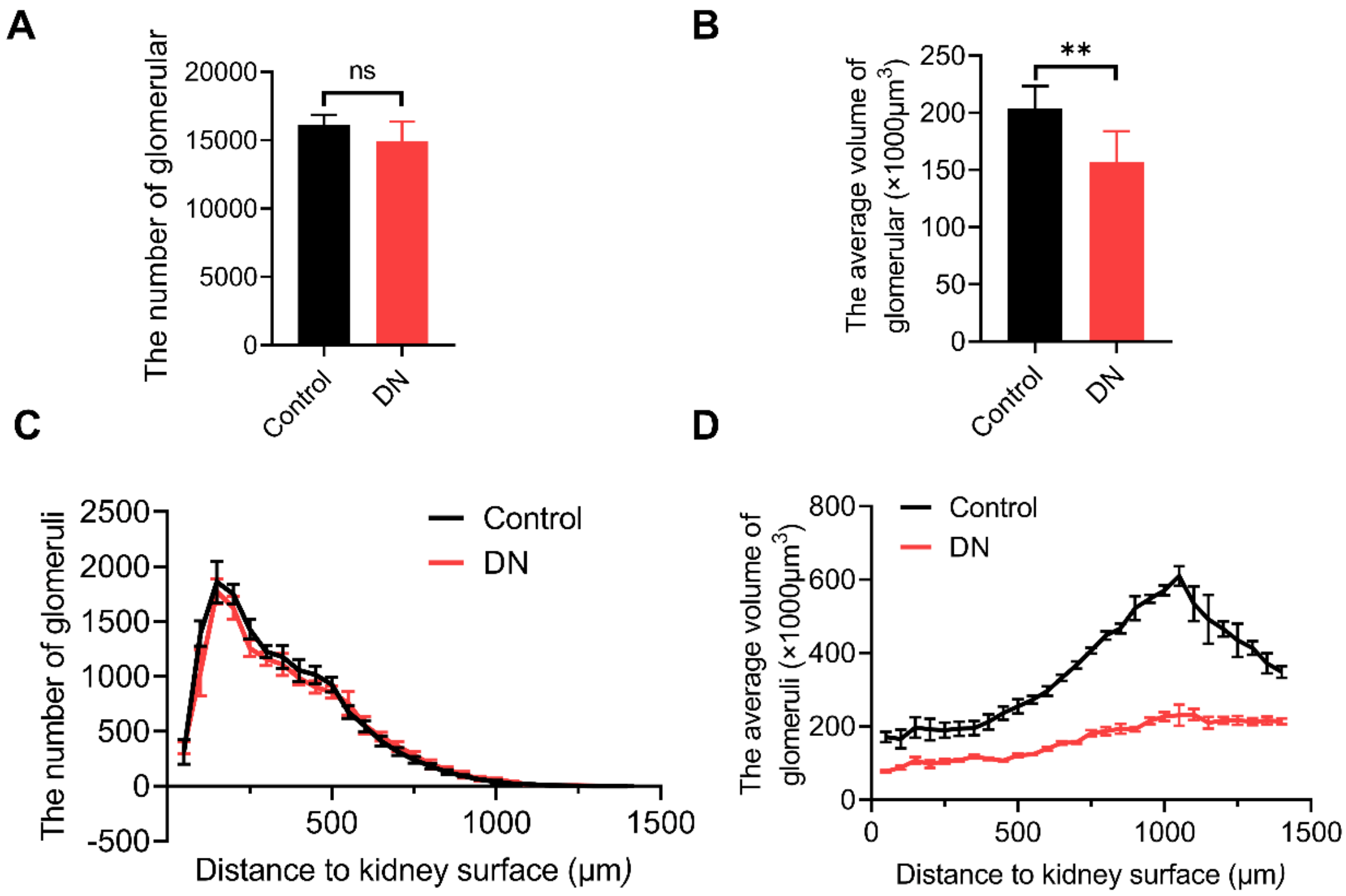

Figure S12. The quantitatively analyze the number (A) and average volume (B) of glomeruli in the Control and DN mice, n = 6 (One kidney per mouse, 6 mice per group). The quantitative determination of the relationships between glomerular number (C), average volume (D), and their specific locations within the kidney of Control and DN mice, n = 6. All values are presented as the mean ± SD. Statistical significance in (A, B) was assessed using an independent-sample t-test. $^{**}P<0.01$, ns: no significance.

Supplementary tables

Table S1. List of dyes for vessels labeling

Name of reagents	Company	CAT #
FITC-Dextran150kDa	Sigma-Aldrich	FD150S
FITC-Dextran2000kDa	Sigma-Aldrich	FD2000S
Evans Blue	Sigma-Aldrich	E2129
Alexa Fluor 647 anti-mouse CD31 antibody	BioLegend	102416
LEL-Dylight488	Vector Laboratories	DL-1174-1

Table S2. List of reagents for tissue clearing

Name of reagents	Company	CAT #
Triton X-100	Sigma-Aldrich	T8787
4% PFA	biosharp	7007171800
N-Butyldiethanolamine	Aladdin	B299095
Antipyrine	Aladdin	A110660
Nicotinamide	Aladdin	N105042
Tetrahydrofuran	Aladdin	T103266
Benzyl Ether	Sigma-Aldrich	108014
Ethyl cinnamate	Sigma-Aldrich	8002380250
Ethanol	KESHI	2021040702
Triethylamine	Aladdin	121-44-8

Table S3. Renal arteries "Surface" steps and parameters

Steps	Parameter	Control	DN	UUO
Imaging processing	Gaussian Filter	√	√	√
	Background substruction	10		
Surface Grain Size	--	4	4	4
Diameter Of Largest Sphere	--	225	202	155
Manual Threshold Value	--	2300	2000	2500
Filter Surfaces	Number of Voxels Img=1 + Manual screening	√	√	√

Table S4. Renal arteries “Filament” steps and parameters

Steps	Parameter	Control	DN	UUO
Algorithm	Autopath (no loops)	√	√	√
Segment Seed Point Diameter	--	10	10	10
Segment Seed Point Diameter Max	--	225	202	155
Segment Seed Point Threshold	Automatic	√	√	√
Segment Classification	ML Filter	√	√	√
Terminal Segment Postfilter	"Length" below 10.0	√	√	√

Table S5. Glomerular “Surface” steps and parameters

Steps	Parameter	Control	DN	UUO
Imaging processing	Gaussian Filter	√	√	√
	Background substruction	√	√	√
Surface Grain Size	--	6	6	6
Machine Learning Training	--	√	√	√
Split	Region Growing Estimated Diameter	20	20	20
Filter Surfaces	"Area" above + Manual screening	6000 um^2	6000 um^2	0 um^2

Supplementary videos

Video S1. Light-sheet fluorescence microscopy imaging of an intact normal mouse kidney processed with EBDE clearing. Red: Renal arteries; Green: Glomeruli. Scale bar: 1 mm.

Video S2. The 3D reconstruction of renal arteries in a normal mouse kidney after EBDE clearing. Scale bars, 1 mm.

Video S3. The 3D reconstruction of glomeruli in a normal mouse kidney after EBDE clearing. Scale bars, 1 mm.

Video S4. The 3D reconstruction and classification of M1−M3 glomeruli in a normal mouse kidney after EBDE clearing. Scale bars, 1 mm.

Video S5. The 3D reconstruction of renal arteries of the normal (Control) and UUO mice after EBDE clearing. Scale bars, 1 mm.

Movie S6. The 3D reconstruction of M1−M3 glomeruli of the normal (Control) and UUO mice after EBDE clearing. Scale bars, 1 mm.

Video S7. The 3D reconstruction of renal arteries of the normal (Control) and DN mice after EBDE clearing. Scale bars, 1 mm.

Video S8. The 3D reconstruction of M1−M3 glomeruli of the normal (Control) and DN mice after EBDE clearing. Scale bars, 1 mm.
